# Use of Technology to Promote Child Behavioral Health in the Context of Pediatric Care: A Scoping Review and Applications to Low- and Middle-Income Countries

**DOI:** 10.3389/fpsyt.2019.00806

**Published:** 2019-11-13

**Authors:** Keng-Yen Huang, Douglas Lee, Janet Nakigudde, Sabrina Cheng, Kathleen Kiely Gouley, Devin Mann, Antoinette Schoenthaler, Sara Chokshi, Elizabeth Nsamba Kisakye, Christine Tusiime, Alan Mendelsohn

**Affiliations:** ^1^Department of Population Health, New York University School of Medicine, New York, NY, United States; ^2^College of Osteopathic Medicine, New York Institute of Technology, New York, NY, United States; ^3^Department of Psychiatry, Makerere University, Kampala, Uganda; ^4^Ministry of Education and Sports, Kampala, Uganda; ^5^Butabika Hospital, Kampala, Uganda

**Keywords:** mHealth, eHealth, pediatric, behavioral health, parenting, framework, health service, low-and-middle-income country

## Abstract

**Background:** The burden of mental, neurological, and substance (MNS) disorders is greater in low- and middle-income countries (LMICs). The rapid growth of digital health (i.e., eHealth) approaches offer new solutions for transforming pediatric mental health services and have the potential to address multiple resource and system barriers. However, little work has been done in applying eHealth to promote young children’s mental health in LMICs. It is also not clear how eHealth has been and might be applied to translating existing evidence-based practices/strategies (EBPs) to enable broader access to child mental health interventions and services.

**Methods:** A scoping review was conducted to summarize current eHealth applications and evidence in child mental health. The review focuses on 1) providing an overview of existing eHealth applications, research methods, and effectiveness evidence in child mental health promotion (focused on children of 0–12 years of age) across diverse service contexts; and 2) drawing lessons learned from the existing research about eHealth design strategies and usability data in order to inform future eHealth design in LMICs.

**Results:** Thirty-two (32) articles fitting our inclusion criteria were reviewed. The child mental health eHealth studies were grouped into three areas: i) eHealth interventions targeting families that promote child and family wellbeing; ii) eHealth for improving school mental health services (e.g., promote school staff’s knowledge and management skills); and iii) eHealth for improving behavioral health care in the pediatric care system (e.g., promote use of integrated patient-portal and electronic decision support systems). Most eHealth studies have reported positive impacts. Although most pediatric eHealth studies were conducted in high-income countries, many eHealth design strategies can be adapted and modified to fit LMIC contexts. Most user-engagement strategies identified from high-income countries are also relevant for populations in LMICs.

**Conclusions:** This review synthesizes patterns of eHealth use across a spectrum of individual/family and system level of eHealth interventions that can be applied to promote child mental health and strengthen mental health service systems. This review also summarizes critical lessons to guide future eHealth design and delivery models in LMICs. However, more research in testing combinations of eHealth strategies in LMICs is needed.

## Background

### Pediatric Mental Health Needs and Service Challenges in LMICs

The burden of mental, neurological, and substance (MNS) disorders accounts for 10%–14% of the Global Burden of Disease (1, 2), and this burden is greater in low-and-middle-income countries (LMICs) because of high rates of poverty, violence, health problems, and inadequate health systems (3, 4). An estimated 171 million young children in LMICs are “off track” in behavioral- and social-emotional development, which places them on the path to MNS disorders (5). Early prevention and intervention strategies focusing on child mental and behavioral health promotion strategies can reduce this burden and its sequelae, but limited mental health prevention and treatment services are available for children and families in LMICs. Although the World Health Organization (WHO) recognizes MNS disorders as a global priority, and MNS disorders are now discussed at the highest-level policy forums devoted to global health and development, solutions for reducing burden remain limited.

Population health is largely influenced by social determinants (6, 7). High child mental health burden in LMICs may stem from inequalities in social status, resource allocation and opportunities, medical and social service access, and the quality of living environments (7–9). Many children in LMICs are living in families with low financial capital and high levels of family stress (e.g., violence, poverty-related stressors, maltreatment) and in communities with poor mental health service, system, and resource. Adversities and stressors experienced by families can undermine positive parenting and child behavioral regulation, which are associated with higher mental health problems in young children in LMICs (10, 11). From a services perspective, children and parents from LMICs are far less likely than families from high-income countries to have access to parenting information, preventive or promotive mental health services, or participate in evidence-based early interventions because of the lack of child mental health resources and systems networks. Therefore, to effectively address children’s behavioral and mental health needs, and minimize disparities in LMICs, solutions that focus on a wide range of individual, family, systems, and service determinants - as well as prioritizing early prevention and intervention - are needed (12–14).

### Rapid Growth of eHealth Offers New Solutions to Address Barriers At Multiple Levels

The rapid growth and widespread of technology has the potential to address child mental health burden in LMICs by offering new solutions for improving health information and supports, service access, and resource challenges. Emerging studies from both high- and LMICs have provided supporting evidence of the potential to transform health services and systems using eHealth (15, 16). eHealth is defined as the use of information and communication technology (ICT), such as computers, mobile phones, communications satellite, patient monitors, and other technology tools for all aspects of health information, services, and integrated systems. mHealth (or mobile-health), a subcomponent of eHealth, is defined as the use of mobile devices (e.g., mobile phones, portable/mobile patient monitoring devices, personal digital assessment devices, and other wireless devices) for individual medical and public health practice (17). eHealth can be tailored to individual needs, provided at low-cost, used to improve distance communication barriers, support training and management, and is a sustainable implementation model (15, 18). eHealth has been recommended by the WHO as a health service-strengthening strategy, and shown to be effective in promoting individual patient health, enhancing family engagement, health knowledge, service access, team communication, and emergency support globally (19).

At the global country-level, since 2005, WHO has initiated the Global Observatory for eHealth (GOe), a joint group effort to support WHO member countries (including LMICs) to adopt digital technologies to improve public health as well as individual health and well-being (20). Much progress has been made in policies and legislation since the establishment of GOe. As indicated in the 2016 report, 87% of WHO member states had one or more national initiatives on mHealth, 58% of countries had applied eHealth strategies, and almost all of those (56%) had initiated eHealth for monitoring and surveillance of maternal, neonatal, and pediatric health (17, 21–23).

At the eHealth programming and intervention level, two recent eHealth scoping reviews also summarize progress of eHealth applications in child health promotion and in LMIC contexts. In the eHealth applications for child health, Barros and Greffin’s (24) review (24) of 119 technology-based, parent-focused interventions found that different formats of web-based applications have targeted parents and aimed at health-related promotion, *via* both prevention and treatment support (e.g., universal preventions and interventions focused on adaptation to and management of chronic/severe acute health conditions). Most of these e-parent interventions were adapted from evidence-based interventions (EBIs), focused on child physical health (i.e., obesity, healthy eating, vaccination, child safety, alcohol/substance use, health service use, oral health, sexual, and reproductive health), and based on studies from high-income countries. The pediatric eHealth Strategies used were also focused on promoting parental self-management, specific parenting skills, or parent support (e.g., social support, providing customized feedback) (24). In the eHealth applications in the LMIC contexts, Bervell and Al-Samarraie (25) reviewed 66 articles to understand patterns of eHealth use across a spectrum of disease and health conditions in Sub-Saharan African (SSA) countries. They found that eHealth has been applied in a range of diseases/health conditions in SSA, including tropical diseases, infectious diseases (malaria, HIV/AIDS), oral health, infant health-related conditions, maternal health-related conditions (antenatal/postnatal care, postpartum hemorrhage), noncommunicable diseases (cervical cancer, blood pressure), and mental health (depression care). In addition, most eHealth strategies were designed for the purposes of disease/condition control and prevention (e.g., reminders toward medical care/activity adherence), population health monitoring and case report, information provision for treatment/prevention (e.g., health information to patients or health workers), data acquisition and patient records management, diagnosis (telepathology, digital radiology tools), training/recruiting/retaining health professionals, or decision-making/referrals (25).

While many eHealth benefits have been reported in the literature to date, there are limitations as well. For example, eHealth has been shown to be more useful for addressing low-intensity, high-frequency behavioral difficulties than high-intensity behavioral difficulties (26). For users with low literacy (e.g., low digital/technology and/or low literacy/educational attainment) or systems with low eHealth technology capacity or resource availability, the limitations would be greater. Additional steps would be needed before applying eHealth in these contexts (27, 28). Furthermore, the benefits of eHealth may not be guaranteed because mixed results are often reported (29). Well-designed, high quality evaluations are needed to better understand the factors and service delivery approaches and contexts that that contribute to of eHealth-related benefits (30).

In sum, the literature thus far suggests that much progress has been made in the development and implementation of eHealth strategies in both child health promotion and in LMICs; however, applications of these strategies in early childhood behavioral and mental health in LMICs remain limited. Given the growth and spread of technology and access, especially given the explosion in digital device ownership and improvements of ICT systems in LMICs (45%–89% with mobile-cellular telephone, 45%–54% with smartphone, 7%–18% with internet access) (20, 23, 31, 32), and the potential of eHealth to address multiple resource and system management barriers, an effort to build on existing evidence and develop new strategies for child mental health promotion is needed.

### The Study Aims

As the first step to inform the development of eHealth (including mHealth) interventions and services for child mental health promotion in LMIC contexts, it is critical to understand and summarize current research and knowledge, especially related to the technology solutions/strategies, core components, and evidence that contribute to effective child mental health promotion. Moreover, to effectively reduce population mental health burden, early prevention and intervention eHealth strategies in children need to be prioritized. Thus, the overall goal of this paper is to address these eHealth knowledge gaps by reviewing related eHealth literature and applications focused on young children. This scoping review focuses specifically on:

1. Providing an overview of existing eHealth applications, research methods, and evidence of effectiveness in child mental health promotion (focused on children of 0–12 years of age) in diverse service contexts.
2. Drawing lessons learned from the existing research about the design strategies to promote usage (or user-engagement in technology use) and evidence of usability, acceptability, and satisfaction (usage patterns, level of engagement, and satisfaction in usage) to inform design, delivery, and evaluation strategies of future eHealth interventions in LMIC contexts.

## Methods

### Literature Review Methods

A scoping review, drawing upon a broad range of applications of technology in medicine, psychology, and pediatric-related literature, was conducted. The scoping review method was applied because it provides a useful initial approach to generate foundational knowledge, and to inform approaches for a future systematic review (33). Therefore, this paper was not intended to be an exhaustive review of the literature, but rather to provide a high-level view of the approaches to the use and evaluation of eHealth strategies for child mental health promotion and prevention. In our scoping review, the five-step method outlined by Arksey and O’Malley (33) was applied. The five steps include: (1) identifying the research question; (2) identifying relevant studies/literature; (3) selecting studies; (4) charting the data; and (5) collating, summarizing, and reporting results.

**Figure 1:**
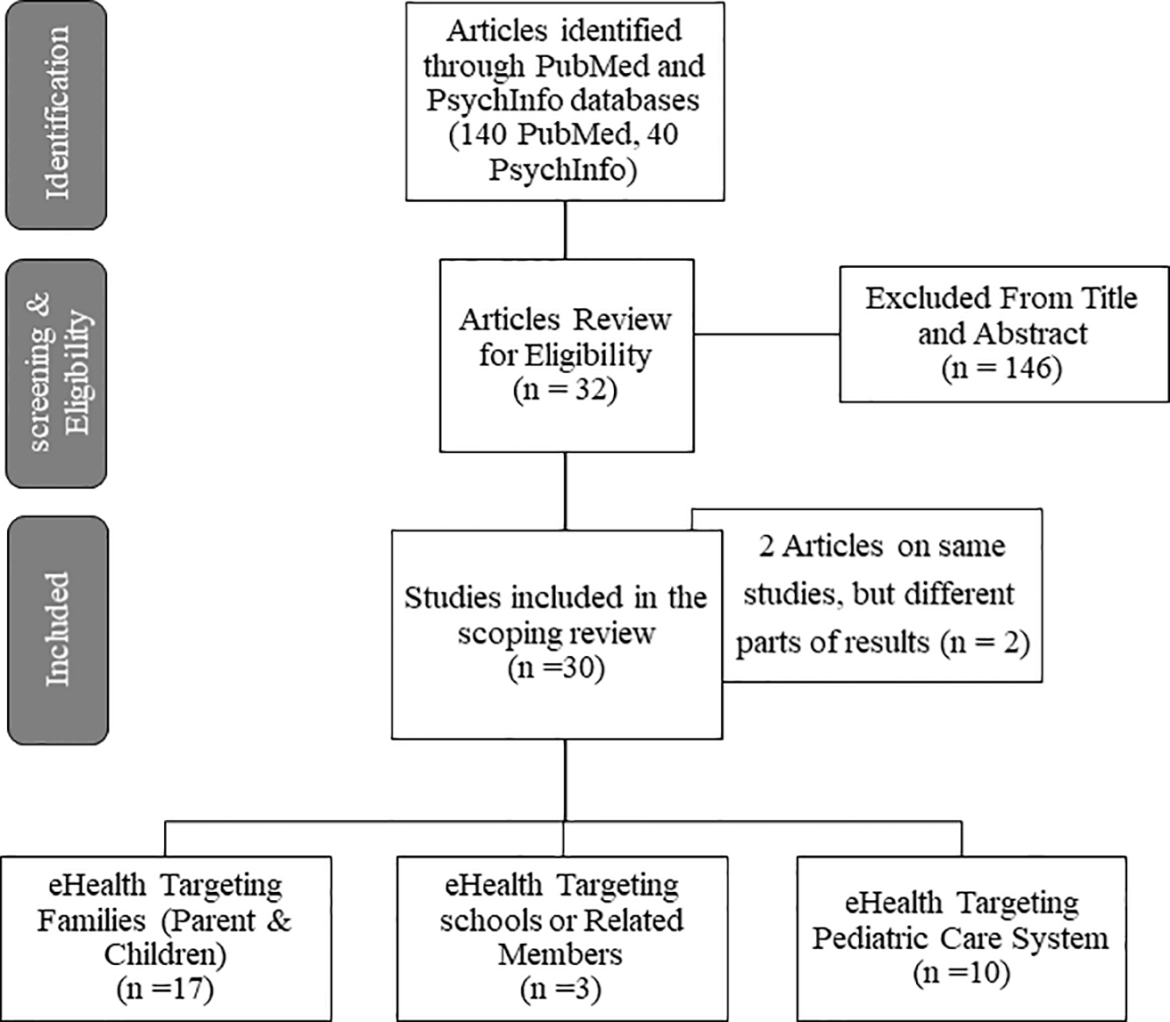
Flowchart of the Selection of Articles.

A comprehensive literature search using the PubMed and PsycInfo databases was undertaken. Literature search terms used in this review are detailed in Box 1 in the **Appendix Supplemental File**. Included papers were critically appraised using the Preferred Reporting Items for Systematic Reviews and Meta-Analyses—Extension for Scoping Review (PRIMSMA-ScR) guideline (34). The overall inclusion criteria of articles for this review included studies that: (1) examined eHealth applications in pediatric behavioral and mental health promotion or intervention; (2) reported either intermediate impacts (e.g., on providers) or direct impacts on child well-being; (3) examined eHealth strategies used in diverse service contexts (i.e., primary care, school, and community contexts); (4) were focused on young children (birth to 12 years; not adolescents); (5) were peer reviewed, published in English, in PubMed or PsycInfo from 2010 to 2018; (6) were not using telehealth or messaging/texting; and (7) were not focused on behavioral health related to childhood obesity and substance/alcohol use. We did not include telehealth in the review because most families in LMICs do not have access to digital tools for videoconferencing, which has been reported to be a more effective child telemental health approach (35). We did not include messaging/texting in the review because evidence suggests effective public health approaches to child mental health intervention require consideration of multicomponent interventions (e.g., including multiple domains of mental health knowledge, skill training, and practice support in parenting/child/health-worker interventions) (36–38). However, existing messaging/texting strategies tend to be used as support or enhancement strategies for interventions, and might be limited in serving as stand-alone mental health intervention strategies in multicomponent interventions (39–41). Finally, many adults and parents in LMICs have low literacy (primary or less than primary school education) and do not have smartphones, which make a messaging approach challenging. **Figure 1** shows the flowchart diagram of the selection of articles. Because this review relied on publicly available documents and, therefore, was exempt from Institutional Review Board determination.

## Results

One-hundred-and-forty peer reviewed articles from PubMed and 40 from PsycInfo were identified. Two duplicated articles were excluded. After reviewing for appropriateness, 32 articles (from 30 eHealth studies) were included for final review. Of these, 2 were scoping reviews, 4 were protocol papers (all using a randomized controlled trial design), and 26 were empirical articles that used a range of designs (i.e., experimental, pre-post comparison, cross-sectional, qualitative, or mixed method designs), and in different phases of eHealth design and testing (e.g., early user-design, feasibility, or efficacy testing phases). **Table 1S** in the Supplemental file documents the charting of review data in detail for the included studies.

Across the articles reviewed, three broad categories of literature about eHealth in child mental health emerged. We synthesized knowledge and lessons learned separately for these three areas: i) eHealth interventions targeting family members; ii) eHealth for improving school mental health services; and iii) eHealth for improving behavioral health service in primary care settings. About half of the eHealth studies (16/30) were conducted in the US, only one study was conducted in LMIC (Brazil), and the remaining studies were conducted in developed countries (13/30, such as Australia (5/30), UK, New Zealand, Netherlands). Regarding the content of eHealth interventions, about half of the eHealth studies (17/30) were adapted from evidence-based interventions (EBIs). The majority of the studies (23/30) reported impacts of the intervention using an experimental design (14/30), and almost all of these studies reported significant positive impacts (21/23).

**Figure 2:**
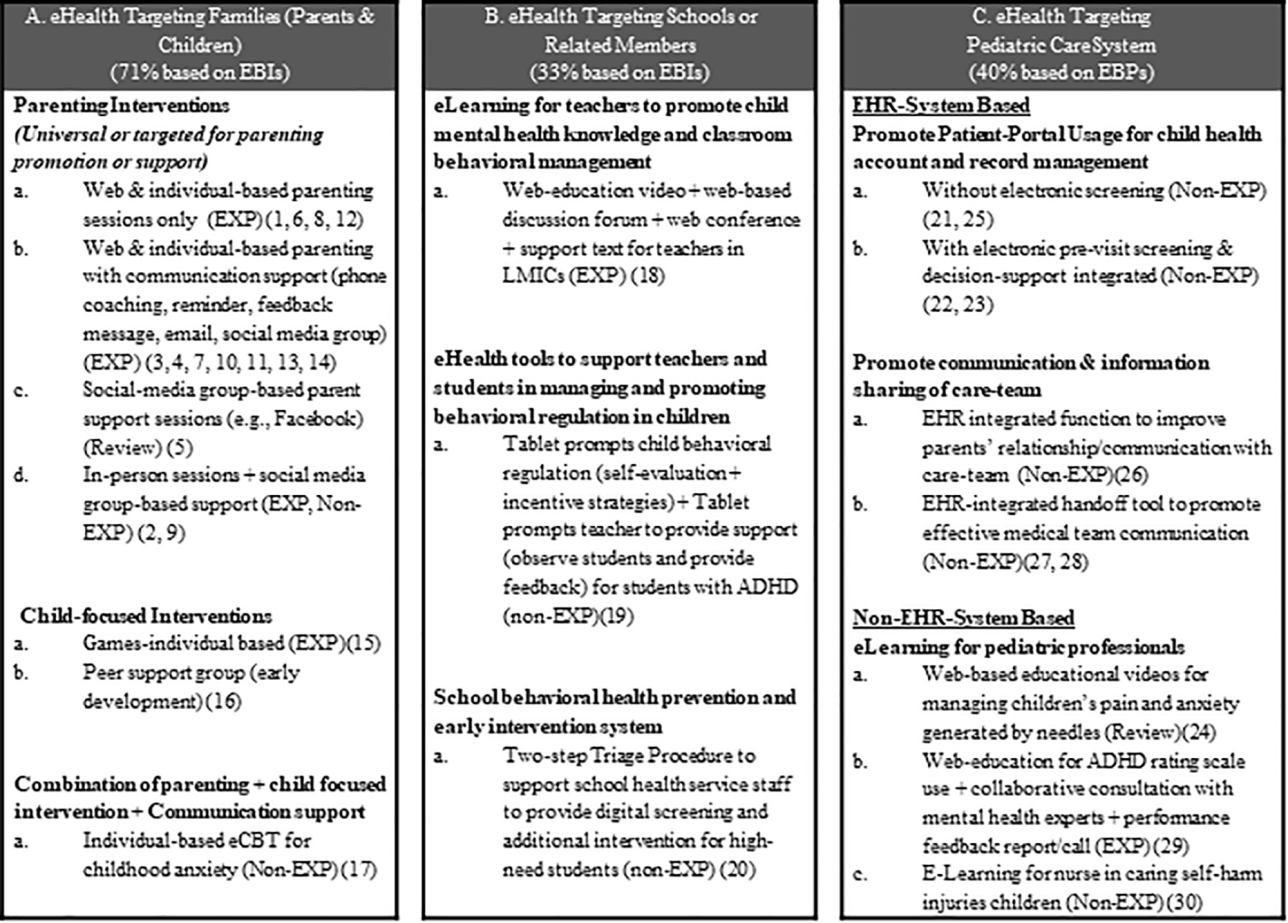
Pediatric eHealth strategies for families, schools, and pediatric care. EXP, Experimental design; Non-EXP, non-experimental design; Numbers included (e.g., (20)) were corresponding study number (not reference number) listed in **Table 1** and **Table 1S**. EBIs, Evidence-based interventions; EBPs, Evidence-based practices/guidelines. Box A summarizes purpose and eHealth strategies applies that target families. Box B summarizes purpose and eHealth strategies applied that target schools. Box C summarizes purpose and strategies applied that target pediatric cares.

**Figure 2** provides a pictorial view that summarizes strategies and ways that eHealth has been applied in these three areas. **Table 1** presents key findings for the 30 eHealth studies that were reviewed (from 32 articles), cataloged by eHealth intervention, country, targets, methods, design strategies, and impact or feasibility evidence (derived from **Table 1S**, the charting of review data in Supplemental file). Below, we summarize findings for the three areas of child behavioral eHealth literature. In each area of eHealth literature, we highlight target users, purpose of the eHealth design and strategies applied, and efficacy/effectiveness evidence.

**Table 1:** Pediatric e-behavioral/mental health literature: study targets, methods, strategy, and impacts for the included articles.

mHealth study/intervention	Country	Targets	Methods	mHealth strategies					Impacts
		Parent, child, provider	EXP, non-EXP, review)	EBI/EBP (Y)	Level of TX (I, G, S)	Parenting digital strategies	Child digital strategies	Provider/system digital strategies	Intervention effect
***Family-Level eHealth Strategies***									
1.Web-Based Parent Management Training for children with conduct problems (42)	Sweden	Parents of 3–12 y/o	EXP (RCT)	Y	I	Web parenting training (7 sessions)			Positive (on P& C)
2. Brief home visit parenting intervention SafeCare + SafeCare-Facebook parenting group for parents at-risk for maltreatment (43)	US	Parents of 0–5y/o	Non-Exp (qualitative)	Y	I & G	3 weekly home visit sessions + 18 Facebook parenting network group			Positive (on P)
3. Stepping Stone web-based TX for promoting parenting knowledge and parent-child relationship (44)	Korean	Parents of 11–16 y/o	EXP (Quasi-Exp)		I	Web parenting training 4 weeks + weekly telephone coaching			Positive (on P)
4. Strongest Families Smart Website (45)	Finland	Parents of 4 y/o	EXP (RCT)	Y	I	Web parenting training sessions (11 sessions) + weekly phone coaching			Positive (on P & C)
5.Online Parent Social Support for parents of children with Special Health Care Needs (CSHCN) (46)	US	Parents of CSHCN	REVIEW (a scoping review)		G	Parent-to-parent support through digital media, particularly social media (Facebook) on emotional and informational support			Positive (on P, especially for aged 18–29 young adults)
6.ezPAREN, online parenting training program (47, 48)	US	Parents of 2–5 y/o	EXP (RCT - Protocol)	Y	I	Web/App-based self-administered parenting (6 modules)			Effectiveness not Yet Report, but adherence and user-engagement were high.
7.Triple P Online Community (TPOC), an online parenting program with social media and gaming features (49)	US	Parents of 2–12 y/o that are highly vulnerable	Non-EXP (pre-post)	Y	I & G	Web parenting (eigth sessions) + social media group support + reward system			Positive (on P)
8. (a)(b) Triple P- Positive Parenting Program- Online Brief- TPOL Brief) (50, 51)	Australia	Parents of 2–9 y/o with mild to moderate conduct problems	EXP (RCT)	Y	I	Web parenting training (5 modules) + Optional technology assisted communication tools (e.g., remainder, summary email)			Positive (on P & C)
9. Parent-Wellness WhatsApp Support Group to promote mothers’ wellbeing of children with autism spectrum disorder (52)	Saudi Arabia	Parents of 26–78 month with Autism spectrum disorder	EXP (RCT)		I & G	4 WhatsApp-based parent psychoeducation & support sessions + 1-face-to-face session			Positive (on P)
10. Cool Little Kids Online Parenting training for prevent child anxiety (53)	US	Parents of 3–6 y/o who are at-risk for Anxiety	EXP (RCT)	Y	I	Web parenting training (8 modules) + Telephone consultation by psychologist when requested			Positive (on P & C)
11. COPING, a universal web-based parenting program to promote positive parenting (54)	US	Parents of 3–8 y/o	EXP (RCT - Protocol)	Y	I	Web parenting training (10 sessions) + automated feedback + online praised message + text message reminders			Not Yet Report
12. Universal internet-based ParentWorks parenting program (55)	Australia	Parents of 2–16 y/o	EXP (RCT - Protocol)	Y	I	Online-based parenting training (8 modules)			Not Yet Report
13. Telephone-Supported Triple P-Online Parenting Program TPOL (TPOLe) for parents with behavior challenge children (56)	Australia	Parents of 1–8 y/o with disadvantage or family difficulty	EXP (RCT)	Y	I	Web parenting training (TPOL) (8 sessions) + weekly telephone consultation sessions for 8 weeks			Positive (on P& C)
14. Parenting Resilient Kids (PRK), a web-based parenting program for child behavior problem prevention (57)	Australia	Parents of primary school-aged children	EXP (RCT- Protocol)		I	Web-based parenting assessment + web-tailored parent feedback report + personalized online modules (up to 12 interactive modules)			Not Yet Report
15. Adventures computer-based game to improve child social skills and mental health (58)	US	7–11 years old with social skills challenges	EXP (RCT)	Y	I		9 interactive online adventure games (include feedback, prompts based on performance)		Positive (on C)
16. Development of a Digital Peer Support Service (DPS) for children coping challenges (59)	US	8–12 year-old cancer survivor	Non-EXP (qualitative)		G		Personas-method (an user-center design method) was used to co-design DPS		DPS contents (3 primary personas for DPS)
17. BRAVE-ONLINE, web-based cognitive behavior therapy (CBT) for childhood anxiety (Primary focused on children, and secondary on parents) (60)	New Zealand	7–15 year-old children with mild to moderate anxiety after a nature disaster	Non-EXP (Pre-post follow-up)	Y	I	Web-based parenting modules (5 for parents of adolescents, and 6 for parent of younger children) + auto-reminder for missing a session + therapist support/weekly contact	Web-based child modules (10 20–45 min sessions) + therapist support/contact		Positive (on P& C)
***School-Level eHealth Strategies***									
18. Web-based Learning Management System (WBIE) for training teachers on child mental health and management (61)	Brazil (LMIC)	Teachers of primary school students	EXP (RCT)		I & S			6 web-education videos (9 hr) + internet discussion forum + web conference + support text	Positive (on T)
19. iSelfControl, web-based application to support classroom behavioral management for students with ADHD (62)	US	Teachers and 9–11 years old children with ADHD	Non-EXP (13 days follow-up dyadic data)		I		Tablet prompts the child to self-evaluate and earn points for adaptive behaviors (every 30 min) + view progress & compare with teacher’s rating	Tablet prompts the teacher evaluate child behaviors (record every 30 min) + view student rating & progress	Positive for some C (for those with high inside)
20. Two-step Triage Procedure for pediatric behavioral health preventive care in primary school (63)	Nether-lands	School health service (SHS) staff serving 4–8 years old students	Non-EXP (Cross-section feasibility study)	Y	I & S			A digital screening carried out by SHS assistants, and only children in need of follow-up were assessed by the SHS doctors or nurses	Positive (on School preventive service)
***Pediatric Care/System eHealth Strategies***									
21.Patient-Portal for Parents in pediatric care: feasibility & usage (64)	US	Parents of young children	Non-EXP (Cross-section feasibility study)	Y	I & S	Parent-portal for child health account, information, and record management		Patient-Portal integrated with EHR system	Positive (on primary care service & P)
22.Comprehensive electronic previsit screener: parent and provider experience and impacts (65)	US	Parents and Primary care physicians (PCPs) of 4–10 years old	Non-EXP (mixed method)	Y	I & S	Parent use of electronic previsit screeners for child behavioral health		Electronic Previsit screening system for parents and PCPs	Positive (on primary care service, P and PCPs)
23. PEDStestOnline, a web-based pediatric screening & clinical recommendation (www.pedstest.com/online) system (66)	US	PCPs and parents of 0–8 years-old	Non-EXP (existing data from 22 sites in 20 states)	Y	I & S	Parent-portal for child health screening (no screening results) and record management		Electronic screening + Parent-Portal with/without integration with EHR system	Impact evidence not reported (Report only patterns of utilization and implementation)
24. Public available Educational Videos for managing children’s pain and anxiety generated by needles (30)	Global	Parents and PCPs of infants and toddlers	REVIEW (a scoping review)		I	Web-based behavioral management strategy videos for parents or PCPs	child pain/anxiety management strategy videos	25 Public Educational Videos from YouTube & Google search	No impact evidence reported
25. eRedBook, a digitized version of EHRs in UK: Implementer and user experience and barriers (67)	UK	School health staff and parents of young children	Non-EXP (Qualitative study)	Y	I & S	Parent-portal for health management (enrolled by public health nurses and health visitors)		Enrolling parents to use EHR-Integrated Parent-Portal system	Negative (Many enrollment barriers; e.g., safety, complexity, literacy, WiFi availability)
26. An EHR-based tool with names, photos, and definitions of treatment team members to increase parents’ accuracy in identifying care team (68)	US	Parents in pediatric care	Non-EXP (pre-post survey)		I & S	Parent use of integrated EHR functions to identify care team members & to build trust		An EHR function to improve parent-care team communication	Positive (on P)
27. eHand-over Tool, an EHR-integrated medical handover tool to improve medical handover between doctors (69)	Australia	Pediatric Providers	Non-EXP (cross-sectional survey)		S			An EHR-integrated tool to standardize and improve both the standard and efficiency/efficacy	Positive (on pediatric care, Dr. satisfaction & workflow)
28.EHR-integrated strategies to increase discharge communication in multidisciplinary team & Impacts (70)	US	Pediatric PCPs & hospitals physicians	Non-EXP (Qualitative study)		S			4 strategies to improve communication: Standardize process; Make it Easy; Eliminate waste; & Incentivize	Positive (on Pediatric and Primary care service)
29. SHARE intervention, a multicomponent distance-learning/quality improvement program to improve PCPs’ use of ADHD rating scale (71)	US	Pediatric care practices and PCPs	EXP (RCT)		S			SHARE includes: Web-based education; Collaboratively consultation with ADHD experts; and Performance feedback reports/calls	Positive (on PCPs and Primary care service)
30. Digital Education Program Development to Train nurse in caring for children with self-harm injuries (72)	US	Nurses in pediatric care	Non-EXP (a participatory approach)		S			e-Learning program for nurse that is sensitive to nurses’ and care recipients’ needs	No impact evidence reported (Report digital tool development process)
**Total**	**16 US; 1 LMIC; 13 Others**	**17 Family; 3 School; 10 Pediatric**	**14 EXP; 14 Non-EXP; 2 Reviews**	**17 Y**	**14 I; 2 G; 3 I+G; 4 S; 7 I+S**	**21 Parents (14 parenting training; 2 parent Support-Groups; 5 EHR-Portal)**	**5 Children (2 educational video; 1 game, 1 behavior regulation, 1 peer support)**	**15 System (3 school system; 10 pediatric system)**	**23 Impact Reported (21 positive; 2 Negative or uncertain)**

TX, Intervention. Level of TX; I, individual family/child-focused; G, family/child group-focused; S, system-focused (school or pediatric care system). EBI/EBP, Evidence based intervention (defined as intervention adapted from EBI that used nondigital approach or digital-based EBI)/Evidence-based guideline (defined as evidence-based practice guideline recommended by professional pediatric society); Y, Yes. Exp, Experimental evaluation (e.g., RCT, experimental comparison, quasi-experimental). y/o, years old. Intervention Effect: P, Parents; C, Child; T, Teacher; PCP, Primary care physicians;

### eHealth Targeting Families

#### Target Users

Most family-level eHealth interventions (for children 0–12 year olds) have targeted parent users (14 parenting-focused studies out of the total 17 family-focused eHealth studies), and fewer directly targeted child users (two child-focused out of 17 family-focused studies) or a combination of parent and child users (1 out of 17). Parents and children were from either community or at-risk samples (e.g., children with behavioral challenges; families with multiple adversity indicators or high stress).

#### Purpose and eHealth Strategies Applied (**Figure 2A**)

For parent-targeted eHealth interventions, most interventions were developed to improve parents’ child mental health knowledge and behavioral management skills/practices through multisession/module and multicomponent interventions (purposes). These types of interventions were usually adapted from existing EBIs (71%). eHealth strategies applied include web-based parenting modules (included 4 to 12 interactive or structured parenting sessions), with (44, 45, 52–53, 54, 56, 57) or without (42, 47, 48, 50, 55) additional e-communication or e-support for parents (e.g., phone coaching, e-reminders, feedback messaging, email, social media groups). There were also some studies focused on parent support interventions (including information and emotion support) and wellness promotion (purpose) for families of children with challenging behavior. For these support/wellness interventions, social media, and group-based e-support strategies (e.g., Facebook, WhatsApp), with or without in-person sessions, were generally applied (43, 46, 52).

For child-targeted eHealth interventions, one study focused on promoting child social skills and mental health (for 7–11 years old) through use of online gaming sessions (including 9 game sessions, with feedback and prompts based on performance). The game components included game goals, rules, game mechanics, and procedures to generalize or transfer game learnings to daily life (58). Another study focused on the development of digital peer support tools by first focusing on content development, using a user-centered and participatory method to design personas (59). Yet other study targeted parent and child simultaneously; this multiuser and more intense treatment intervention focused on children with mild to moderate anxiety with an adaptation of cognitive-behavioral therapy (CBT) to a digital approach (with e-training modules and digital communication support) (60).

#### Efficacy/Effectiveness Evidence (**Table 1** and **Table 1S**)

Among the reviewed parent-focused eHealth studies that had outcome data (11 studies), 7 were evaluated using experimental designs (70%). All 11 eHealth parent-focused interventions reported positive impacts on parenting, and six interventions also reported positive impacts on child mental health (based on short term or less than one-year follow-up period). Among the reviewed child-targeted eHealth studies that had child outcome data (2 studies), one used an experimental design, and all child studies (with 9–10 eHealth sessions) showed positive impacts on child mental health (58, 59).

### eHealth Targeting School Mental Health Services

#### Target Users

Among the identified school-focused eHealth literature that promotes young children’s mental health (3 studies), interventions targeted teachers (1/3 studies), school health personnel (1/3 studies), or students who had behavioral or mental health challenges (with teachers’ involvement; 1/3).

#### Purpose and eHealth Strategies Applied (**Figure 2B**)

eHealth in school contexts has been applied to strengthen school mental health resources or improve school mental health care. For the eHealth that targeted teachers, the intervention was designed to train teachers on child mental health and classroom behavioral management (purpose). It used a web-based learning management system (WBIE) approach, including web-education videos (6 modules), online discussion forum, web conference and support text messaging, to support teachers’ child mental health education and practices (61). This study was conducted in an LMIC (Brazil). For the eHealth that targeted school health personnel, the intervention was designed to entail a two-step triage approach to support school behavioral health services. In Netherlands, community-based school-health professionals (e.g., physicians, nurse, and health assistants) visit schools a few times a year for routine child health and behavioral health assessments. For students identified with problems or needs, additional services are provided. The eHealth two-step triage strategy was designed to provide preassessments (a digital screening questionnaire) to primary school students by trained school health assistants, as well as a built-in follow-up decision support function that allows the school health team to be notified to follow need-identified students (63). For the eHealth that targeted students with behavioral challenges, the intervention was design to improve ADHD students’ self-regulation in classrooms by involving target students and their teachers in the behavioral observation-feedback loop (purpose). The eHealth strategy was to use a digital tool (iPad-based) to: (i) prompt students to self-evaluate their own adaptive behaviors (e.g., following instructions/rules, staying on task), (ii) earn rewards (points added or subtracted from their account); and (iii) view teacher feedback and compare self-ratings with teacher ratings of their behaviors (62).

#### Efficacy/Effectiveness Evidence (**Table 1** and **Table 1S**)

 Two of the school-focused eHealth interventions reported positive impacts on teachers or school health services (61, 63). Only the study conducted in the LMIC (for teacher education about child mental health and behavioral management) was evaluated using an experimental design (61). The ADHD digital-tool intervention only benefited students with high insight (62), indicating the importance of considering user characteristics in eHealth design and implementation.

### eHealth Targeting Pediatric Care Settings

#### Target Users

Most eHealth in pediatric care focused on pediatric professionals, adults who care for children, or users of EHR systems (i.e., parents, pediatric care providers/PCPs, pediatric care team).

#### Purpose and eHealth Strategies Applied (**Figure 2C**)

For eHealth interventions that targeted pediatric professionals or adults who care for children, the interventions were usually designed for education purposes. For example, e-learning strategies and web-education models/videos might be applied to educate/train adults or professionals to screen or care for children with mental health related problems (e.g., ADHD, self-harm, anxiety) (30, 71, 72). The e-learning strategy might be combined with other e-collaborative/consultation or performance feedback communication functions (e.g., report or call) to provide additional support (70).

For eHealth interventions that targeted EHR users, two groups of studies were identified. One group of studies focused on promoting patient-portal usage to improve preventive mental health screening, record management for patients/parents (64, 67), and/or decision support for providers (e.g., integrate screening/decision notifications for providing additional services) (65, 66). For screening and patient record management, web-based or EHR-based previsit screening and e-account management strategies have been applied. These e-strategies were implemented through navigator assistance or self-serve account setup. For provider decision support, integrated screening/EHR strategies (e.g., with automatic scoring, alter-notification/prompt for action when at-risk case identified, and automated decisions that is built into the clinical workflow) have been applied.

The other group of EHR studies focused on promoting communication and information sharing among the pediatric care team. This could be either improving communication between patient and care team (68) or improving communications (standard, procedure, information format) among medical care team members (69, 70). eHealth strategies such as e-demonstration and integration of standardized activities/practice guidelines/forms/prompts with EHR processes have been applied.

#### Efficacy/Effectiveness Evidence (**Table 1** and **Table 1S**)

Among the three reviewed e-learning studies (targeting pediatric professionals or adults who care for children), only one study evaluated the outcome using an RCT design (70). This study found that distance-learning that integrated web-based education, collaborative consultation, and performance feedback for PCPs on child mental health screening could increase PCPs’ use of behavioral screening tools in pediatric care.

Among the EHR studies that focused on promoting patient-portal use, most showed positive evidence (2 of 3 studies), using a nonexperimental design. Parents showed improvement in use of EHR-portal or screening tools and positive pediatric care experience (64, 65). However, one study showed negative findings and challenges while implementing a patient-portal due to technology issues (e.g., web access issues, poor technology literacy in users) (67).

Among the EHR studies that focused on promoting communication and information sharing, all studies (three of three) reported positive impacts and user experience (e.g., increase use of e-communication tools/procedures, improvement in communication, care workflow) (68–70). However, none of these were evaluated using experimental designs or had reported impacts on child mental health outcomes.

### eHealth Design Strategies for User Engagement and Usability

To draw lessons learned from the existing eHealth research on design strategies for engaging users in eHealth interventions (or design strategies that maximize products’ usability, accessibility, and target users’ needs) to inform future eHealth development in LMIC contexts, we synthesize findings from studies that discussed or provided evidence related to user engagement/user-centered strategies, usage patterns, level of engagement, and satisfaction of eHealth interventions. Twenty-one studies out of 32 studies that we reviewed provided these user engagement results and discussion; therefore, these articles were used for research synthesis.

Based on the available articles, we grouped user-engagement design strategies and usability evidence/lessons into four areas of eHealth applications. These include user-engagement strategies and usability lessons related to: i) e-parenting intervention design, ii) health worker eLearning/e-training design (i.e., eLearning strategies for pediatric providers, school health staff), iii) designing integrated e-screening and e-decision-support tools in primary care, and iv) designing workflow integrated e-communication/collaboration tools. We summarize findings in the sections below. **Tables 2A, B** also summarize key lessons from our analysis. User-engagement strategies marked with ** in **Table 2** were those that we believe are relevant to LMIC contexts.

**Table 2:** eHealth user engagement strategies, usage patterns and acceptability evidence.

(a) eHealth user engagement strategies (** Lessons also relevant to low- and middle-income countries (LMICs))	
eHealth in Contexts	User-Engagement Strategies
eHealth in Parenting Intervention Context	**Parent recruitment** *via* social media vs. general practices**: Parents recruited from general practices tend to stay on to the program for longer time than parents recruited from social media (given established social bonds or therapeutic alliance relationship) (44) Strategies for addressing Technology literacy**: For families with low technology literacy or not use technologies on regular-basses, including one introduction session in the beginning of parenting program to help sign-up a private group account, and demonstrate online tool functionality can be helpful (43) Privacy/Safety strategies**: Parents prefer respectful communication and information sharing. Contents that they or other parents share should be careful chosen and appropriate. Parents felt comfortable sharing parenting information with an anonymous group (43) Motivators or incentive strategies to promote e-parenting technology use**: (i) promoting relationship tides between parents and the online communities/social networks (44, 56); (ii) including a social network group in e-parenting intervention (social network as a sharing community); (iii) inclusion of Facebook “events”, (iv) including incentive approaches (e.g., raffle tickets, win prizes, gaming approach/achieving badges) to promote participation motivation, (v) having more contact with interventionist through social media channels (group messages, regular reminders, instant charts) (43, 49) Learning Engagement for Parents**: i) parents prefer working in group that have more similarities between parents themselves and other parents (e.g., similar-aged children, geographic locations); ii) parents commented that it would be helpful to see more examples of the skills that other parents are participating (from interventionist or other parents) (43); iii) web-based eLearning with some forms of consultation or learning support (from earlier cohort of parents or implementers) can promote parents’ skill learning and total number of session completion rate (49, 53, 56)
eLearning in Provider/System Context (for PCPs or School Teachers)	Web vs. offline eLearning strategies**: For staff e-training, both web-based interactive education (WBIE; including a discussion forum to interact with consultants, and a web conference with a child psychiatrist) and the video-based education (TVBE; including receiving text, but not in-person connection) can be effective eLearning approaches (e.g., both types of eLearning had more nonstigmatized concepts than the control). However, the WBIE interactive approach was more effective than the TVBE noninteractive approach (e.g., in knowledge gain, fewer stigmatized concepts/opinion). Results suggesting adding a **discussion forum** and **web conferencing** can have more knowledge gain, but not on changing attitudes (61). A multicomponent eLearning strategy**: a three-component distance-learning can be effective, which includes web-based education [3 15-min modules], collaboratively consultation with child mental health experts, and performance feedback report/calls (71) Co-developing training/educational materials** is an important way for designing educational resources that included a strong patient voice, meet nurse learning needs, and ensures the content is relevant, appropriate and sensitive to both the recipient of care and those responsible for its delivery (72)
E-Screening & E-Service Decision Support (in School Health Service or Primary Care Context)	Integration of two-step triage procedure is a good and acceptable way to set up preventive behavioral health care in schools**. The two-step triage includes a digital screening conducted by health assistants (task-shifting to community health workers) and a referral or additional service conducted by health professionals for those screened positive (task-sharing) (63) Patient portal enrollment and engagement strategies (in primary care)**: **i)** Use a navigator to demonstrate the patient portal to parents can increase the sign-up rate and address parent’s technology literacy barrier (64); **ii)** Provide computers in the waiting-room and using waiting-room attendant (e.g., gap year students or retirees paid close to minimum wage, who can help interview families with limited literacy or can be charged with entertaining children, modeling appropriate adult-child interaction, implementing Reach and Read) that address family technology access and technology literacy gaps to improve enrollment and portal use (56%) in comparing to a reminder option (which give parents an appointment reminder card, including information on how to log-in to PEDS online and request to complete screens before the next scheduled visit) (44%) (66); **Web- vs. On-site kiosks approach for setting up Patient Portal**: Many parents who sign up for patient portal also wanted to have the access of patient portal (for their child) *via* on-site kiosks (64) **Integration of Previsit screening with EHR Decision Support**: *Parents like this approach when*: **i)** the digital approach is easy to use; ii) the screening highlighted the areas of concern that the doctor needed to touch on/discuss during the well-child care visit; **iii)** the screening questions relevant to their needs/questions (which remind them areas of concerns they may discuss with providers or areas of child health issues that they have not thought of; iii) completing the screener in advance that improve visit efficiency. Providers like the approach when**: **i)** reducing the workload; **ii)** a summary report was provided to discuss with parents; **iii)** comprehensive screening questions were used (e.g., inclusion of mental health and other nonphysical health issues on the screener) that eliminating the need for the PCP to take the time to assess these issues (65, 67)
E-Communication in Care Team Collaboration Context	e-Strategies for trust-relationship between patients and care team members**: including names, photos, and definitions of treatment team members (e.g., role) can promote parent ability to correctly identify the care team, trust relationship, and care satisfaction (68) Four strategies to enhance pediatric care team communication**: 1) development of standard process for e-Handover; 2) reduction of overproduction and defects by “Making it Easy to Follow the Standard” and by providing resident/care team education; 3) eliminating waste in wait and search times (*by improve use of EHR for communication*, e.g., supplying care team members with providers’ phone number and prefer methods of contact); and 4) aligning the incentive with those performing the work (70)
(b) eHealth Usage Patterns and Acceptability Evidence
eHealth in Contexts	eHealth Usage Patterns and Acceptability
eHealth in Parenting Interventions	**Safe-Care Facebook Social Support group**: Use Frequency: Families with computers at home were more likely to check Facebook regularly (e.g., 3 times weekly) than families that relies on public facilities (e.g., computer in library; e.g., participate in some weeks). Parents commented on the content that they enjoyed viewing (of others’ postings) within the group including parenting resources, links to websites, and supportive comments to and from other parents (43) **Web-PMT (7 self-paced parenting sessions)**: 66% completed all 7 sessions, 22% completed 3–6 sessions, 16% completed fewer than 3 sessions. 69% families with two parents participated together (42) **ezPARENT (6-sessoin Parenting program self-administrated modules)** (in the US): On average, parents spent 37.2 min per module (SD = 22.2); the mean number of program visits was 13.6 (SD = 8.6; range 2–49). Average length of time per visit was 14.1 min (SD = 17.1). Participants completed on average 82% of the modules (out of 6 total modules) (47). **Triple P Online Community (TPOC) (8 online modules + social media + incentive in the US**): online modules were access through numerous channel, such as agency computer lab (70%; home computer (54%), cell/smart phone (51%), work or school computer (33%), iPad or tablet (31%), friends’ computer (21%), free WiFi (restaurant, 20%), public library computer (16%). The complete rate for the entire 8-module program was 36–51% (higher rate when smartphone is available, in later cohort/with support from earlier cohort)(71) **Triple P Brief (TPOL Brief)-(Self-directed 5 modules, with optional technology assisted communication tools** (e.g., text prompts reminder, send module summary *via* email): 62% completed at least the recommended minimum dose (introductory module + one exemplar module), 53% completed 3 or more, 45% completed 4 or more, 40% completed all 5, 13% completed introductory only, 25% did not completed any. Average module completion time was around 2 hours for the introduction and 45min for the exemplar modules which is longer than expected, indicating that parents were explored optional extra material. 88% rate the program as good, and 77% were at least satisfied with the program. Parents with high disagreement over parenting were less likely to complete minimum dose of intervention (50, 51) **TPOL-with weekly Telephone support (8 weekly online module + telephone consultation)**: parents in the TPOLe condition completed significantly more modules and higher satisfaction than directed TPOL (M = 5.62 and 3.25; 47% vs. 23% completed all 8 modules). Mean module completion time was 63 minutes. TPOLe group participated in 4.36 (SD = 2.53) clinical telephone support session on average. Average call duration was 24 minutes (SD = 8). There was a significant correlation between the number of telephone consultations and number of online modules completed (56) **Online-Cool Little Kids parenting program (8 online modules + telephone consultation when requested)**: Online program use was lower than the high attendance rates generally observed for the group parenting program when delivered through a university research clinic. Only 1/3 of parents attended most sessions (53) **BRAVE-ONLINE (eCBT; 10 online sessions for children, and 5–6 online sessions for parents)**: On average, by 6-month follow-up, children and adolescents had completed 5.9 of 10 sessions (SD = 2.9) and parents had completed 4.56/6 (SD = 1.7)(child parent program) and 2.95/5 (SD = 1.9) sessions (adolescent parent program); 35% and 41% had completed all their sessions at the time of the follow-up (60)
eLearning for Providers or Teachers	For **school-based e-Training** (for promoting teacher mental health in LMICs), the **attrition rate was high across all group**: 31% for WBIE and 52% for TVBE (might be due to high rates of teacher absenteeism and time unavailability due to high workloads). If participated, the impact was positive after receiving training (61) **For Pediatric care-based eLearning**: 79% completed all 3 educational presentations. There were on average two phone, email, or in-person consultations with ADHD experts per month. 57% clinicians participated in at least one performance feedback call. Use of Care Assistant were more frequent in intervention (36% use at least 5 times, and 19% use at least 10 times) than in Control (31% use at least 5 time, and 15% use at least 10 times). Intervention clinicians who participated in at least one performance feedback call were more likely to send out parent rating scales than intervention clinicians who did not participate (relative difference of 14.2 percentage points (71).
E-Screening & E-Service Decision Support (in School Health Service or Primary Care Context)	**Schools using the 2-step triage procedure to set up preventive child behavioral health service**, they provide more accessible service to students (measured by increasing utilization of/contacts # with school health service professionals than the schools not using 2-step triage. **This approach is also perceive as an more appropriate approach to support children with special needs** (63) **Implementation of Pediatric Screening by Providers (use of PEDStestOnline** (www.pedstest.com/online): PEDS (evaluation of developmental status) was most commonly used (100%), followed by PEDS : DM (evaluate developmental milestones) (41%) and M-CHAT (Autism screening)(21%). Use of the M-CHAT spiked around 18months of age and remained high in the months surrounding 24 months of age. The screening use decreased ager 3 years of age (66). **Staffing for Implementing Pediatric-Screening**: Receptionists/medical technician stations were often served as the point for dissemination clipboards/screening measures (at check-in). Next, skilled nurses often in charged with entered parents’ responses into PEDS Online, offering an interview if forms were incompleted (clarified parents’ comments and answers to items)or if evidence of limited literacy was present (66) **Patient Portal Usage (for screening and health record management)**: In the US, less than 50% clinics implemented web-portal. For the clinics that implemented, % family used portal varied (range of family reached was 35%–100%), and only 10% use previsit screening. English speaking parents were more likely to use the online portal and screening thank non-English speaking families, but no difference in presence or absence of porta use due to parents’ level of education or poverty. Among the users, about 70% of parents reported that they planned to use the patient portal again for their child (after sign-up). Since activation, median use was 0.8 times per month. A two-year tracking among disadvantaged populations found 81% of patients who activated their accounts accessed the portal twice or more (64) **Parent use of previsit screening tools**: Parents indicated high acceptability of the screeners. Nearly 90% trusted the security of the screener, and 87% thoughts their answers would be confidential; 92% thought the screener was a good way to ask routine questions, 95% were comfortable with the mental health questions, and 89% thought the screener helped with sharing of concerns (65)
E-Communication in Care Team Collaboration	**After implementation of EHR-Care team introduction system**, parents showed improved rate for correctly identifying care team physicians (71% s 28%). Most parents (79%) and care-team members (87%) also reported that subjects’ ability to identify care team members impacted their satisfaction and trust relationship (68) **Communication compliance and process significantly improve after implementation of e-handover communication Tools**: 15 out of 19 services have improved communication; satisfaction increase from 17% to 678%–7%) (69); 81% of PCPs were followed and confirm that e-handover communication was received (70); the Tools significantly simplify the care process (measured by 68% of PCP reported significantly reducing paper work time, and reduction of redundant data from 52%) (69)

#### User-Engagement Strategies and Usability in e-Parenting Intervention Design

In engaging parents to use e-parenting interventions (i.e., e-parenting in individual or group format), several strategies related to parent recruitment, technology literacy, privacy/safety, motivation to use e-parenting technology, and engagement in learning were identified as critical. For example, recruiting parents from traditional face-to-face practices would be a better recruitment strategy (e.g., with better retention rate) than recruiting parents from social media given personal contacts promoting more social bonds and therapeutic alliance relationship (44). To address technology literacy challenges in e-parenting technology use, including an introduction session to help parents sign-up/set up an account and learn about e-tool functionality can be a helpful strategy (43). To promote parents’ participation frequency in use of eHealth tools/modules, including incentive strategies (e.g., use raffle tickets, win prizes, achieving badges, use of social network, promote online communities) and additional communication strategies (e.g., contact with the interventionists, use group messages, regular reminders, instant chat, technical support) are critical for improving effectiveness and usability (43, 49). To support parents learning and behavioral changes, eLearning with some forms of consultation or learning support (e.g., from implementers or other experienced parent-peers) and integrating parents’ preference of learning styles/activities (e.g., working in groups with similar aged children, geographic locations) can bolster parenting behavioral changes or support (49, 53, 56).

##### Usability

Eight out of 14 parent-focused interventions reported eHealth usability. Three key lessons were gleaned from the findings: (1) frequency of eHealth usage would be higher when the accessibility of the digital devices is high and easy to use (in format that matches with target users’ life style), and when resources/contents were relevant and more interactive (43) (49); (2) full completion rate for all modules/session increases (35%–66%) when the eHealth interventions are well designed (using short modules [15–30 minutes/per module], includes five or more sessions, and includes multiple communication supports (e.g., 20 minutes/per contact) (42, 47, 49–50, 51, 56, 60); and (3) when social network/e-community and optional technology-assisted communication tools were included, the likelihood to complete a minimum dose of intervention and user satisfaction can generally increase (50, 51, 56).

#### User-Engagement Strategies and Usability in Health Worker eLearning/e-Training Design

In training health workers (i.e., school staff, pediatric providers), one important lesson gained was that eLearning using web-based and offline video approaches were found to be equally effective in training. This is especially relevant to LMIC contexts, given challenges in internet access in many regions. Furthermore, interactive web-based education that also included other learning support (e.g., a discussion forum, opportunities to interact with consultants/child psychiatrists, web-conferencing, performance feedback calls/report) can further promote health workers’ skill learning, behavioral changes, and session completion rate (61, 71). For content design, codeveloping E-educational materials/content between health workers and targeted children/families can better meet health workers’ needs, and ensure the content is relevant, appropriate and sensitive to both the recipients of care and those responsible for its delivery (72).

##### Usability

E-learning in LMICs has been found to be more challenging than e-Learning in developed countries, with a lower reach/usage rate in LMICs (i.e., 48%–70% reach in LMIC-Brazil vs. 79% in developed countries). Reasons for low reach include high rates of staff absenteeism, workloads, and time constraints, all of which indicate the importance of addressing contextual barriers in eLearning designs (61, 71). When performance feedback sessions were provided (to offer in-person consultation/support to answer trainees’ questions about e-Learning content or to promote use of skill after eLearning), just over half (57%) of trainees participated in at least one feedback call. Those who used feedback calls were more likely to adopt the skills learned in eLearning (71). Findings suggest the importance of standardizing feedback sessions as part of the eLearning delivery models.

#### User-Engagement Strategies and Usability in Designing Integrated e-Screening and Electronic-Service-Decision-Support Tools for Primary Care

To promote provider and parent use of an integrated e-screening and decision support tool (with triage function), user-engagement strategies targeting parent users and care provider users (including physicians, coordinated implementers) need to be considered. Efficient strategies that have been identified to promote patient-portal use/enrollment and use of previsit screening for their children include: use of navigators/waiting-room attendants (to demonstrate, assist sign-up, support parents who have low literacy), providing computers/on-site kiosks in waiting rooms, using reminder cards (with specific requests, instructions and information for using e-screening), keeping digital functions easy to use, making screening questions relevant to parents’ needs, and summarizing areas for further attention (or highlighting areas for further discussion with health providers) (64–67). To promote provider uptake and use of integrated e-screening and decision support tools in pediatric care (for behavioral health promotion), providing training on the digital tool use (with clear explanation of functions and clinical workflow processes) and providing tools that have multiple benefits in meeting providers’ needs (e.g., reducing workload, improving clinical efficiency, a summary report to discuss with parents, including comprehensive screening questions that eliminating the need for providers to take the time to assess) are critical to consider in design and delivery models (63, 65, 67).

##### Usability

Use of a parent portal for routine pediatric care is relatively new in pediatric behavioral health care settings in the US. Results of the PEDStestOnline study (that included 79 providers across 20 states, with data from 20,941 children ages birth to 8 years) indicated that 30% of sites implemented a web portal, and the rate of family enrollment varied by providers (reach 35%–100% families). Additionally, only 10% of eligible families used a previsit screening. The rate of uptake could depend on enrollment approaches. English-speaking parents were more likely to use the screening and online portal than non-English speaking parents, but there were no differences in portal use by parents’ level of education or poverty (66). For clinics that provided computers in the waiting room, the uptake for portal use was higher (56%) than the clinics that used the appointment reminder approach of enrollment (44%). For those parents enrolled, about 70% reported that they planned to use the portal again for their child, and 81% continued accessing the portal two years after initial enrollment (66). Parents also reported high acceptability and satisfaction (e.g., about 90% trusted the security of the screener, 92% thought the screener was a good way to ask routine questions, 95% were comfortable with the mental health questions, and 89% thought the screener helped with sharing of concerns) (65).

#### User-Engagement Strategies and Usability in Designing Workflow Integrated e-Communication/Collaboration Tools

Applying collaborative and team-based care models to integrate behavioral health service in routine primary care is a recommended clinical practice guideline for pediatric care, especially for addressing the needs of special or high-risk pediatric populations (73–76). Although eHealth research in this area is limited, it is encouraging to see some new research. Based on two studies included in this scoping review, several user-engagement strategies have been suggested to enhance pediatric care team e-communication. These include (1) developing standard processes for communication and integrating these into e-communication tools; (2) providing care team education and making the standard of e-communication easy to follow; (3) improving use of EHR/e-tools for communication (e.g., supplying care team members’ photos, expertise/roles, contact numbers, and preferred methods of contact in the e-tools); (4) providing incentives for those performing the work/or using the standard (68, 70).

##### Usability

The usability of e-communication tools and/or standardized e-handover tools were high. Parents and primary care-team members report improvement on their care satisfaction and trust relationship with the care team (79% and 87%, respectively) after use of the tools (68). For e-Handover communication tools, 79% of care teams reported improvement in team communication, increased satisfaction of care provided to pediatric patients (17% to 67–87%) and clinical efficiency (e.g., 68% reported a significant reduction of paperwork time and/or simplified care processes) (69, 70).

## Discussion

The purpose of this paper is to address knowledge gaps in applications of eHealth to promote young children’s behavioral and mental health, as well as to understand how eHealth has been applied to broader dissemination of child mental health EBIs. The scoping review method was applied to generate a high-level overview of the eHealth strategies used and evaluated in child mental health contexts. A total of 32 articles (from 30 studies) were selected for this review. Through this review, eHealth applications (what purposes and problems to be solved) and digital- and user-engagement design strategies (how to best design) that have been applied and demonstrated efficacy/usability/effectiveness in child mental health promotion and prevention were identified and described. eHealth strategies that apply to parents, children, schools, and primary care practices were also identified. Based on our review, we noted several areas where additional eHealth research is needed to develop better approaches to support users in child mental health promotion and prevention both in global and LMIC contexts. In this section, we present research-practice implications for eHealth research that are relevant to LMIC contexts as well as discuss current limitations of the pediatric eHealth field.

### Implications for eHealth Development and Design in LMICs

Four eHealth applications and design lessons can be drawn from this review. First, in identifying eHealth strategies to promote child mental health, three key eHealth design principles were consistently identified across three areas of eHealth applications (parenting, school, and primary care intervention). These include **i)** core intervention strategies that promote target users’ child mental health knowledge and management skills can be developed and adapted from existing EBIs; **ii)** in promoting e-session/module participation (e.g., increasing adherence, preventing attrition), one or more supporting strategies that motivate participation, promote relationship-connection, or address technical challenges must be in place (e.g., using in-person contact, telephone contact, social media group access, gaming/incentives, automated email, messaging strategies); and **iii)** in promoting skill practice and behavioral change, consultation, and learning/supporting strategies that match target users’ preferences and needs must also in place to ensure the practice changes (e.g., providing consultation support using a group or individual approach).

Second, in designing eHealth that fits LMIC contexts, two lessons can be drawn from the review: **i)** given limited web/internet availability in LMICs, the design of eHealth strategies should use mixed approaches by combining offline video-based psychoeducational learning/training (individual or group-based) with mHealth or/and face-to-face support strategies. This might be a feasible model given evidence that either web-based or offline, video-based education approaches can result in promising positive outcomes (61), and that the combination of mHealth and in-person support strategies (in group or individual formats) can be useful and highly acceptable for users with different levels of family risk or child mental health problems (42, 49, 56); **ii)** given that evidence has shown similarity in human behavioral change mechanisms across ethnic groups and high- and LMIC populations (14, 77), lessons learned from high-income country-based literature in user-centered design and user-engagement strategies (described in **Table 2**) are likely to be relevant and applicable to populations in LMICs. However, eHealth design and strategies may need to be tailored to local contexts and to be more thoroughly evaluated.

Third, the potential for applying eHealth strategies as cost-efficient approaches to address healthcare barriers is high. This scoping review demonstrates that a body of pediatric health research has successfully transported nondigital EBIs to eHealth formats, and has demonstrated the feasibility and effectiveness of this approach in high-income country contexts (24). There are also studies showing evidence of the cost-benefit of eHealth interventions in LMIC settings (78). These findings are encouraging as more EBI literature emerges.

Fourth, from our review, we noted ways that eHealth can be conceptualized and applied as multicomponent/multicontext digital strategies/solutions to promote child mental health. For eHealth to be effective, many eHealth interventions need to integrate multiple components, such as including strategies to promote mental health knowledge, practice and skills. Also, many eHealth interventions need to consider and address needs across multiple contexts, such as considering both family/home (e.g., a child’s home), service provider contexts (e.g., primary care, schools), and linkage of both contexts. The multicontext concept is particularly useful in current mental health intervention service development research given a growing emphasis on linking mental health services from home to communities and health systems, and applying collaborative and team-based care models to integrate mental/behavioral health service in routine primary care and community settings (including the Mental Health Gap Action Program/mhGAP mental health service model suggested by the WHO, which suggests the application of collaborative, task-shifting and task-sharing implementation strategies in the provision of mental health services in LMICs) (73–76).

**Figure 3:**
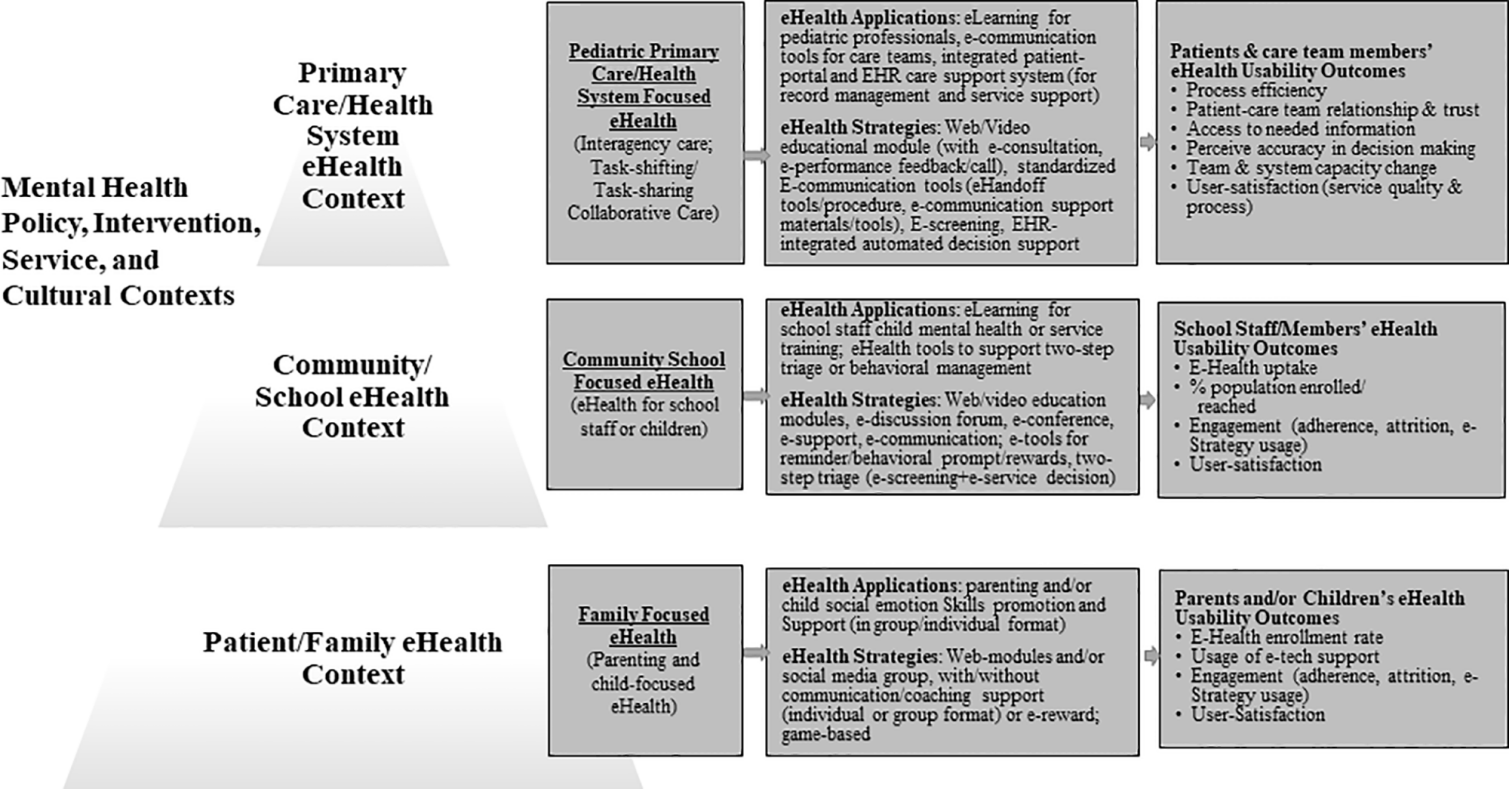
A multicontext framework for child mental and behavioral eHealth. The proposed framework is developed from this eHealth literature synthesis. This framework considers implementation contexts and processes that described in the WHO (2009) Service Pyramid Model (79), and Proctor et al.’s (80) implementation framework.

As an attempt to provide a working framework to guide future eHealth dissemination and implementation research, we summarize our lessons learned in an integrated multicontext framework for child mental and behavioral eHealth (**Figure 3**). The framework highlights key e-Health applications and strategies at individual/family, school system, and primary care contexts that can be applied to promote child mental health, as well as highlights eHealth usability/implementation outcomes to be considered and measured in future research in order to advance eHealth research and practice.

### Limitations and Directions for Future Research

As a result of this review, we have identified several gaps where additional eHealth research is needed. One, eHealth capacity or system strengthening has rarely been discussed in the eHealth literature or in child mental health research. Given limited eHealth research in LMICs, building eHealth research and system capacity are needed (using the approaches suggested above). eHealth capacity building at the policy/governmental, academic, community, and technology-sector levels will can develop the needed workforce and expertise in designing user- and population-centered eHealth solutions. Two, well-designed high-quality evaluations, such as applying experimental designs to eHealth intervention outcomes studies, are still lacking. More research is needed to map out specific components/approaches of eHealth and associated benefits that can be applied to future development of behavioral health interventions. Three, to advance eHealth research, methodology, and measurement tools for assessing eHealth contexts, target users’/agencies’ readiness for eHealth intervention (e.g., technology resources, technology literacy), and eHealth usability and implementation outcomes (e.g., user-engagement level, user-centeredness, usability) need to be further developed. Having better measurement tools and consistent methodology will facilitate cross study comparisons and better mechanism testing research. Four, although telehealth and messaging/texting is not included in this review, given growing applications of these strategies in middle-income countries (81), we suggest a separate scoping review to better understand the applications and stand-alone/unique impacts of these approaches on child health and/or mental health. It will be especially important to consider the impact and applications of telehealth/messaging in those populations that have better access to these eHealth strategies as well as better literacy.

## Conclusion

As child mental health issues continue to require complex health service and healthcare policy solutions, it will become increasingly important to develop eHealth solutions that consider multiple contexts and integrated multicomponent solutions. This paper provides several new directions to address eHealth programming and methodological gaps related to eHealth research. The scoping review and framework not only provide guidance on how eHealth-related contexts and implementation/usability outcomes can be conceptualized, but also how eHealth mechanisms can be integrated into more robust implementation designs. As has been reiterated, more research is needed to elucidate both cross-setting and multisetting eHealth strategies and mechanisms. In particular, systematic and long-term follow-up research will strengthen understanding of eHealth strategies to advance eHealth implementation effectiveness, sustainability, and system and population-level mental health outcomes.

## Author Contributions

K-YH, DL, JN, S-Che, KG, DM, AS, S-Cho, EK, CT, and AM contributed conception and design of the study. K-YH, DL, JN, and S-Che were involved in the acquisition, analysis, and interpretation of data. K-YH wrote the first draft of the manuscript. K-YH, DL, S-Che, KG, AS, and AM contributed to manuscript writing. All authors contributed to manuscript revision, read, and approved the submitted version.

## Funding

This research was supported by grant NIH/NCATS 1UL1TR001445 from the National Institutes of Health (NIH), and R21 MH110001-01 and U19MH110001-01 from the National Institute of Mental Health (NIMH). The reviews and opinions expressed in this article are solely those of the authors and do not necessarily represent the views of NIH. This review relied on publicly available documents and, therefore, was exempt from Institutional Review Board determination.

## Conflict of Interest

The authors declare that the research was conducted in the absence of any commercial or financial relationships that could be construed as a potential conflict of interest.
