# Supplementary material for: Use of Technology to Promote Child Behavioral Health in the Context of Pediatric Care: A Scoping Review and Applications to Low- and Middle-Income Countries
Source: Front Psychiatry. 2019 Nov 13;10:806. doi: 10.3389/fpsyt.2019.00806 (PMC6865208; doi:10.3389/fpsyt.2019.00806)
Supplement: Supplementary file 1 [file DataSheet_1.pdf]

APPENDIX. THE SUPPLEMENTAL FILE

Literature search terms and papers included in the review are listed below. Box 1 lists search terms used across database searches. Table 1S lists papers and findings by applications of mHealth in 4 areas of pediatric behavioral health literature (for 0-12 years children): mHealth in (i) parent-targeted intervention, (ii) child-targeted intervention, (iii) school system, and (iv) primary-care system. A brief summary of results for each paper is also provided in Table 1S. The reference number correspond to the article number listed in the manuscript Table 1.

BOX 1. Search terms and restriction applied in literature search

Search terms

1. (((mobile health) OR (digital health) OR (mhealth) OR (ehealth) OR (medial technology and health)) OR (electronic health records OR (online) OR (web-based) or (social media))) AND
2. (((mental health) OR (behavioral medicine) OR (behavioral AND Pediatric) OR (parenting AND intervention) OR ((mental health or behavioral health) and (communication or promotion or prevention))) AND
3. ((primary care) OR (provider) OR (community health worker) OR (schools)) AND
4. (2010/01/01 [Date-Publication] : 2018/12/31 [Date-Publication]) AND
5. (“English and humans”[Filter] AND “child<birth to 12 years>” [Filter]) AND
6. (NOT (obesity) NOT (message, text) NOT (adolescent) NOT (abuse, alcohol) NOT (abuse, substance) NOT (telehealth))

Table 1S. Summary Results for Pediatric Behavioral mHealth Research Articles for Children 0-11 years old (or below 12 years old)

Author name and year	mHealth Aims, Methods, and Targets	mHealth Feature (Technology Approach)	Outcomes
**Parent-targeted mHealth Intervention Literature**			
1 **Enebrink, et al., 2012 (42)**	• **Aim**: Evaluate a web-based parenting-training program for children with conduct problems<br>• **Methods**: RCT (n=104, TX/C=58/46) (assessment at pre-post TX and at 6-month follow-up)<br>• **Targets**: parents of 3-12 years old in Sweden	• **The Web-based PMT-treatment**: The TX was theoretically based on social learning theory/cognitive-behavior therapy. It was developed from a Swedish Parent Management Training (PMT) program (group-based; teaching parents parenting strategies for handling behavior problems, implementing behavior modification programs and improving the quality of the child-parent relationship), with good outcome in recent studies and adapted for Internet and intervention at individual level, compared to the group PMT. The Internet-PMT covered similar themes as other PMTs do, but had fewer sessions (7 sessions, compared to 11 sessions for the PMT-group) and was self-paced. The Internet-PMT focused more on positive parenting, communication, positive reinforcement, whereas information and work about response-cost/ punishment of problematic child behavior was summarized to one session. PMT-group also includes instructions for a meeting between therapist, parent and the child's schoolteacher, which was not arranged through the Internet-based PMT.	• At post-treatment, children whose parent(s) had received the TX showed a greater reduction in conduct problems. Between group intent-to-treat effect sizes (Cohen's d) on Problem scales were .42-.72 (study completers .66 and 1.08). Parents in the TX group reported less use of harsh and inconsistent discipline after the treatment, as well as more positive praise. Effects on behavior problems were maintained at 6-month follow-up.<br>• **Engagement/Usage**: 66% completed all 7 sessions, 22% completed 3-6 sessions, 16% completed fewer than 3 sessions. 69% families with two parents participated together
2 Edwards-Gaura, et al, 2014 (43)	• **Aim:** Study feasibility of a combination of brief home-visit parenting TX sessions (SafeCare) + SafeCare-Facebook social network parenting group for child maltreatment prevention<br>• **Methods**: Two pilot feasibility/user studies<br>• **Targets:** parents of 0-5 years old in the US	• **Study 1: At-risk parents' technology use patterns** (n=12): study parents use of smartphones and use of online social networking technologies (Survey)<br>• **Study 2: Pilot mHealth Feasibility** (n=3): 3 single African American (AA) mothers completed a brief behavioral parent training SafeCare (Parent-Child Interaction/PCI components only) that included a social networking enhancement component. Intervention (TX**)** includes 1 introduction session (including sign up a private **SafeCare Facebook group** & demonstration of online tool functionality), 3 weekly home visit sessions to conduct PCI module (a *Behavioral Parent Training Program*), and 18 weeks of Facebook group outside of the sessions (include daily communications by the interventionist about SafeCare-related skills, posting of favorite parenting websites, links to articles, and positive feedback about other posting)	• *Technology use:* 100% AA mothers own a cell phone (92% use daily); 66% had smartphone (92% with phone-internet, 67% send picture & 33% send video using phone); 75% reported owning a computer; 89% had reliable internet access at home; and 67% used the Internet daily<br>• *Social Networking & Facebook use*: 100% with Facebook Account (75% like Facebook, 25% use daily, 50% use weekly, 25% use monthly)<br>• Three parents participated in the intervention all reported improvement in parent-child interaction skills and a positive experience participating in the social networking-enhanced SafeCare components<br>• **Usage**: Families with computers at home were more likely to check Facebook regularly (e.g., 3 times weekly) than families that relies on public facilities (e.g., computer in library; e.g., participate in some weeks). Parents commented on the content that they enjoyed viewing (of others' postings) within the group including parenting resources, links to websites, and supportive comments to and from other parents

**3 Choi et al., 2016 (44)**	• **Aim**: Evaluate a 4-week web-based parenting TX Stepping Stone to address parent-child relationships<br>• **Methods**: A cluster quasi-experimental two-group design (6 schools in 4 cities; N=TX/C:47/46)<br>• **Targets**: Parents of 11-16 years old in Korea	• Comparing parents participating in the **'Stepping Stone' web-based intervention** (a 4-week program that helps Korean parents acquire knowledge, communication and conflict management skills).. The weekly session in both groups have same procedure (after logging in → consent page → pre-training survey → watch a 20-min-long media file that parents could stop and watch again at any time → 3 weekly homework assignments (for Stepping Stone group only)	• After completing the 4-week Stepping Stone program, participants in the intervention, compared to the control group demonstrated preliminary evidence of improved parental knowledge. There were also group differences in the level of knowledge sustained 4 weeks after the post-test. Parents in the intervention group also showed increased parental self-efficacy, parent–child communication, and satisfaction with parent–child relationships, and decreased parent–child conflict, and parental stress
**4 Sourander et al., 2016 (45)**	• **Aim: S**tudy efficacy of Strongest Families Smart Website (SFSW), an web-assisted parenting training<br>• **Methods:** RCT at a primary health care clinic (n=464; TX/C=232/232)<br>• **Targets:** Parents of 4 year-old in Finland	• Parents of 4-year-old children were randomized into **the Strongest Families Smart Website (SFSW)** intervention group or an education control (EC) group<br>• The **SFSW** is a 11-session Internet-assisted parent training program that included weekly telephone coaching (45 mins). Two booster-coaching sessions (to review the skills) were included around 7 & 10 month. The themes of the 11 sessions are: (1) noticing good behavior, (2) spreading attention around, (3) ignoring whining and complaining, (4) transitional warnings and when-then statements, (5) planning ahead in the home, (6) the behavior chart, (7) planning ahead outside the home, (8) working with daycare, (9) timeout, (10) problem-solving relapse prevention, and (11) putting it all together<br>• Control received access to education website + education telephone call	• At 12-month follow-up, improvement in the SFSW intervention group was significantly greater compared with the control group on the following measures: CBCL/1.5-5 externalizing scale (d=0.34), internalizing scale (d=0.35); 5 of 7 syndrome scales, (aggression: d=0.36; sleep: d=0.24; withdrawal: d=0.25; anxiety: d=0.26; emotional problems: d=0.31; Inventory of Callous-Unemotional Traits callousness scores: d=0.19; P = .03); and self-reported parenting skills (d=0.53)
**5.DeHoff et al., 2016 (46)**	• **Aim:** To review evidence of the information and support needs of parents of children with special health care needs (CSHCN) and to determine whether **online social support** can serve as an avenue for learning and empowerment for parents<br>• **Methods:** A **scoping review** & interviews of CSHCN parents and key-informants<br>• **Targets:** parents of CSHCN	• ***Review Rational***: Parent Support is needed for CSHCN. Hospital and health care providers are increasingly communicating digitally. Family support literature suggested that 4 kinds of supportive actions that can help people deal with a crisis or chronic stress and allow them to cope, learn, and even grow during their challenges. This support can be *emotional* (being there), *instrumental* (doing things), *informational* (sharing knowledge and resources), or *appraisal* (helping individuals to see their stressors with more confidence in their ability to cope).<br>• A scoping review (of articles published after 2010) to identify the challenges, coping mechanisms, and support needs among parents of CSHCN, and the **reach and effectiveness of digital technologies** with these families and health care providers. Interviews were also conducted by professionals serving parents of CSHCN	• Review and interviews suggested that parents best learn the information they need, and cope with the emotional challenges of raising a CSHCN, with support from other parents of CSHCN, and that young parents in recent years have most often been finding this parent-to-parent support through digital media, particularly social media, consistent with the theory of online social support.<br>• Evidence also shows that social media, particularly Facebook, is used by nearly all women aged 18-29 years across racial and socioeconomic lines in the United States<br>• Parents of young CSHCN experience significant stress but gain understanding, receive support, and develop the ability to care for and be advocates for their child through parent-to-parent **emotional and informational** social support. **Online social support is most effective with young adults of childbearing age**, with social media and apps being the most useful within the theoretical framework of social support.
**6.Breitenstein, et al., 2016, 2017 (47, 48)**	• **Aim:** evaluate ezPARENT an mHealth parenting training TX<br>• **Methods:** RCT-Protocol (n=312)<br>• **Targets:** parents of 2-5 year-old from primary care	• **ezPARENT** is a digital adaptation of the group-based Chicago Parent Program (CPP; Breitenstein, et al., 2015). The CPP is a self-administered and downloaded on an android tablet. It include 6 modules that teach EBI strategies (2 on positive parenting, 3 on behavior management skills, and 1 on stress management and problem-solving skills.	• **Effectiveness outcome**: Not yet report<br>• **Patterns of use (engagement) and adherence** (based on TX group data): On average, parents spent 37.2 min per module (SD=22.2); The mean number of program visits was 13.6 (SD=8.6; range 2-49). Average length of time per visit was 14.1 min (SD=17.1). Participants completed on average 82% of the modules.
**7. Love, et al., 2016 (49)**	• **Aim:** evaluate feasibility of adding social media and gaming features efficacy of	• **Triple P Online Community (TPOC)** is a online-based Triple P—*Positive Parenting Program* (EBI with 8 online module) that fit vulnerable young adults prefer + *Social media* (encourages peer support by allowing users to share and	• Results showed reductions in child behavioral problems, reduced lax/permissive and over-reactive parenting, and decreased parental stress. No effects were found for parental

	a web-based parenting intervention and support TX, Triple P Online Community (TPOC)<br>• **Methods**: pre-post assess (N=155)<br>• **Targets**: parents of 2-12 year-old that are highly vulnerable parents in US	read program work and "star" each other's points; social sharing with anonymity) + + Gamine features (incentivize the practice of positive parenting strategies through a reward "badge" system + decrease stigma by allowing participants to create a virtual identify which promotes peer support).	confidence, attributions, or depression and anxiety (which were in the normal range at baseline). Positive effects were maintained or improved at 6-month follow-up. The participants engaged in the online community and valued its flexibility, anonymity, and shared learning.<br>• **Engagement & Usage**: Online modules were access through numerous channel, such as agency computer lab (70%; home computer (54%), cell/smart phone (51%), work or school computer (33%), iPad or tablet (31%), friends' computer (21%), free WiFi (restaurant, 20%), public library computer (16%). The complete rate for the entire 8-module program was 36-51% (higher rate when smartphone is available, in later cohort/with support from earlier cohort
**8.a.Baker et al., 2017 (50)**	• **Aim:** Study efficacy of a brief online parenting program (Triple P-Positive Parenting Program-Online Brief; TPOL Brief)<br>• **Methods:** RCT (n=200)<br>• **Targets:** parents of 2-9-year-old with mild to moderate conduct problems in Australia	• **TPOL Brief is a five-module** low-intensity parenting intervention that aims to promote the use of *positive* parenting strategies and reduce child behavior problems. This is a level 3 intervention, *targeted parents of children with mild to moderate conduct problems* in specific problem areas. Users receive personal log in details to the TX and complete the program by themselves at their convenience.<br>• Parent eligible criteria were: 1) with a 2–9-year-old who had elevated levels of child behavior problems; 2) parents identified at least one of four topics covered in the program (i.e. disobedience, fighting and aggression, going shopping, self-esteem) as an area of concern; 3) access to a computer and broadband Internet connection; and 4) ability to read English at Year 5 level.	• At 8-week post-assessment, parents in the intervention group displayed significantly less use of ineffective parenting strategies and significantly more confidence in dealing with a range of behavior concerns. These effects were maintained at 9-month follow-up assessment. A delayed effect was found for child behavior problems, with parents in the intervention group reporting significantly fewer and less frequent child behavior problems at 9-month follow-up, but not at post-assessment. All effect sizes were in the small to medium range. Consumer satisfaction ratings for the program were high
8.b.Baker & Sanders, 2017 (51)	• **Aim:** Study TX use patterns and predictors of use for a brief online parenting program (TPOL Brief)<br>• **Methods:** Using existing data from a TPOL RCT (RX sample only)<br>• **Targets:** parents of 2-9-year-old in Australia	• Participants were 100 parents who receive **TPOL Brief** in Australia (from Baker et al., 2017 article (see above)	• ***Web module use pattern***: 62% completed at least the recommended minimum dose (the introductory + 1 additional exemplar module); 53% completed ≥ 3 module, 45% completed ≥4 modules, and 40% completed all 4 modules. Average module completion time was around 2 hours for the introduction and 45min for the exemplar modules which is longer than expected, indicating that parents were explored optional extra material. 88% rate the program as good, and 77% were at least satisfied with the program. Parents with high disagreement over parenting were less likely to complete minimum dose of intervention<br>• ***Predictors for completing minimum dose***: Younger child age and lower parental disagreement over parenting pre-TX predicted completion of the recommended minimum dose of the TX<br>• ***Who benefit from the online TX*** *(by studying predictors of behavioral change)***)**: higher baseline levels of child behavior problems, older parental age and more intense conflict over parenting pre-intervention predicted greater improvement in child behavior at 9-month follow-up. Improvement in parenting was predicted by higher pre-intervention levels of ineffective parenting.
**9. Hemdi & Daley, 2017 (52)**	• **Aim:** Study efficacy of a WhatsApp web-based psychoeducation TX that promote mothers' wellbeing	• The intervention consisted of one face-to-face session (60 min) and four virtual sessions (30 min each) delivered using **WhatsApp**.<br>• The intervention was developed as a guided self-help intervention in line with the main principles and recommendations of NHS Good Practice Guidance on	• Parental mean total stress score was significantly reduced in mothers in the Tx and maintained at follow-up. Mean scores for all Parent Stress Index subscales were significantly

	of children with autism spectrum disorder (ASD)<br>• **Methods:** RCT (n= TX/C: 34/33)<br>• **Targets**: 26-78 months ASD children in Saudi Arabia	the use of self-help materials within Increasing Access to Psychological Therapies IAPT services. TX content includes 1) what is autism? 2) stress, 3) managing behavior, 4) mood, 5) resources for families	reduced at T2. Also, a significant reduction in maternal depression post intervention was maintained at follow-up.
**10.Morgan et al. (2017) (53)**	• **Aim:** Test efficacy of an web-based parenting program for child anxiety<br>• **Methods:** RCT (N=433)<br>• **Targets:** 3-6-year-old at-risk for anxiety disorders in the US	• Online TX was adapted from the **Cool Little Kids (CLK) parenting group** program (6 group sessions delivered by psychologist to parents of 3-6 year-old) to prevent anxiety disorders in young children who are at risk<br>• **Online-CLK** TX include 8 interactive modules providing strategies that parents can implement with their child to manage their child's avoidant coping, reduce parental overprotection, and encourage child independence. Parents were provided *telephone consultation* with a psychologist when requested.	• The TX group showed significantly greater improvement over time in child anxiety symptoms compared to the control group (d=0.38). The TX group also showed greater reductions in anxiety life interference (ds = 0.33-0.35) and lower rates of anxiety disorders than the control group (40% versus 54%). Parents also reported better confidence, and satisfied with the training. There were minimal effects on broader internalizing symptoms or overprotective parenting<br>• ***Program adherence***: Online program use was lower than the high attendance rates generally observed for the group parenting program when delivered through a university research clinic. Only 1/3 of parents attended most sessions
**11.Owen et al. (2017) (54)**	• **Aim:** Test efficacy of a web-based universal parenting program for parents with an interest in positive parenting.<br>• **Methods:** Protocol paper for a RCT (TX/C ratio=2/1)<br>• **Targets:** 3-8-year-old in the US	• The **online universal parenting TX** is a 10-week online parenting program to promote positive parent-child relations by teaching core social learning theory principles that encourage positive child behavior (e.g., use of praise & reward). Features of the online parenting program include automated feedback based on individual performance, online praise messages for spending time with their child, text message reminders to access the next session and multiple-choice quizzes to test knowledge. The program also enables the tracking of individual usage data, including the number of log in, time spent on each page and the number of chapters completed	• Not yet report
**12.Tully, et al., 2017 (55)**	• **Aim:** Test efficacy of a universal online, father-inclusive parenting intervention<br>• **Methods**: Protocol paper for a RCT<br>• **Targets**: parents of 2-16 year-old in Australia	• **The *ParentWorks***: is based on the Integrated Family Intervention for Child Conduct Problems. Previous studies have evaluated this intervention in different delivery formats, and has been found to be effective in reducing child externalizing problems. The TX was originally developed only for parents of children with conduct problems, and has been modified to be suitable for a broader community sample of parents who may have more general concerns about parenting and child behavior.<br>• **The new *Online-ParentWorks***: can be completed via the internet using a computer, tablet, or mobile phone. The program comprises video presentations of 8 interactive sequenced 'modules', five of which are compulsory. Each video module is approximately 20–30 min in duration. Participants work through the program at their own pace, and depending on which modules they choose, they are able to complete the program in a minimum of three weeks (or four weeks, if they elect to complete Module 5)	• Not yet report<br>• The study will examine the effectiveness of ParentWorks for reducing child externalizing problems and improving parenting, as well as to explore the impact of father engagement (in two-parent families) on child outcomes.
**13. Day & Sanders, 2018 (56)**	• **Aim:** Test the effect of two versions of web-based parenting intervention Triple P Online delivered with varied levels of support	• Triple P Online (TPOL) is a Web-based variant of the Triple P—Positive Parenting Program. It includes eight-module of online behavioral family intervention, based on existing Level 4 Triple P interventions.<br>• ***Self-Directed TPOL (TPOL):*** Multimedia videos are utilized extensively to teach skills and demonstrate strategies, in combination with interactive activities, downloadable resources, and a dynamically generated workbook for tracking progress through the program. Optional technology assisted	• *Short-term effect*: Self-directed TPOL participants showed short-term TX effects, including reductions in overall negative parenting (d=.39-.26) and frequency of child behavior problems (d=.66), while TPOLe led to greater improvements in negative parenting (d=.61-.73) and intensity of difficult child behaviors (d=.93). Participants in the TPOLe were also more likely to complete modules (5.62 vs 3.25; or

	• **Methods**: RCT (N=183) (3 arms: *self-directed Triple P Online (TPOL)* (n=57), *Telephone-supported TPOL (TPOLe)* (n=66), and wait-list control (n=60)<br>• **Targets**: parents of 1-8 year-old with at least one area of disadvantage or family difficulty in Australia	communication tools are embedded (e.g., text prompts to remind parents to try a strategy, send module summaries to a partner via e-mail). The program actively encourages the user to set and review goals throughout, while later modules encourage users to identify high-risk situations and combine strategies and principles discussed earlier in the program into a cohesive prevention plan.<br>• The ***Telephone support TPOL (TPOLe)*** provides up to eight practitioner support session. Parents were encouraged to complete one online module and one telephone consultation each week for 8 weeks (with flexible process and option for reschedule calls). Telephone consultation includes (a) checking that the parent had successfully logged in to the program and/or completed the next module; (b) asking the parent to set an agenda for the session, (c) reviewing module content, (d) reviewing goals or practice tasks from the prior session, (e) discussing agenda items, and (f) discussing an adherence plan if the parent had not engaged with the program since the last consultation.	47% vs 23% completed all 8 modules) and reported greater program satisfaction. Parents in the TPOLe participated in 4.36 clinical telephone support session on average (SD=2.53).<br>• *At follow-up* (longer-term effect), 50% of outcomes for the self-directed condition were significantly better than the control, while 94% of outcomes were significantly better than the control in the practitioner-supported condition. Overall, the impacts are better for TPOLe than TPOL on parenting (d=1.06 vs 0.4), child problem behaviors (d=.70-1.28 vs .52). Parents in the TPOLe also showed lower depression, conflict with partner, and higher confidence (d=.36-.81).<br>• ***Engagement & Usage patterns***: Parents in the TPOLe condition completed significantly more modules and higher satisfaction than directed TPOL (M = 5.62 and 3.25; 47% vs. 23% completed all 8 modules). Mean module completion time was 63 minutes. TPOLe group participated in 4.36 (SD=2.53) clinical telephone support session on average. Average call duration was 24 minutes (SD=8). There was a significant correlation between the number of telephone consultations and number of online modules completed. This research highlights values of support (even minimal) can improve effective engagement (module completion rate), satisfaction, and enhance outcomes.
**14.Fernando et al., 2018 (57)**	• **Aim:** Test the effect of *Parenting Resilient Kids*, an online parenting program for child problem prevention<br>• **Methods**: Protocol paper for a RCT (N=340, TX/C ratio: 1/1)<br>• **Targets**: Parents of primary school children in Australia	• The ***Parenting Resilient Kids (PRK)*** is an online parenting program that aims to reduce family-based risk factors and enhancing protective factors through increased positive interactions between parent and child. The intervention group will receive the PRK consisting of a *feedback report* on parenting behaviors and up to *12 interactive online modules* personalized based on responses to the parent survey.<br>• The active-control group will receive a standardized package of online educational materials about child development and wellbeing.<br>• The trial website is programmed to run a stratified random allocation sequence (based on parent gender) to determine group membership.	• Not yet report
**Child-targeted mHealth Intervention Literature**			
**15.Sanchez et al., 2017 (58)**	• **Aim:** Study efficacy of *Adventures*, a computer-based game to improve social skills and mental health in children with social skills deficits<br>• **Methods:** RCT (N= TX/C:33/36)<br>• **Targets:** age 7-11 years with social skills challenges in the US	• The program, **Adventures**, translates a proven in-person intervention into a nine-episode interactive online adventure game that provides opportunity for knowledge acquisition and skill practice. Children's experience in the game varies based on their previous performance. The game provides feedback, hints, and prompts based on performance and requires skill mastery for progression. The program we developed into Adventures is Social Skills Group Intervention (SSGRIN), an in-person intervention that has been used by thousands of students for over 20 years, and results in significant improvements in social–emotional skills and mental health<br>• Children in the immediate treatment condition completed the game at home over the course of 9 weeks	• Children who played Adventures improved significantly more from pretest to posttest than children who did not play the game in social literacy, social anxiety, bullying victimization, and social satisfaction.

16.Warnestal et al., 2017 (59)	• **Aim:** Applied child personas (a user-centered design) in development of a Digital Peer Support Service (DPS)<br>• **Methods:** Applied a personas-based process to design a digital TX<br>• **Targets:** childhood cancer survivors (8-12 year-old) in the US	• DPS, a peer support program, is needed to help children handle the physical, mental, and social challenges associated with cancer survival and remission. A personas-based process was applied in design of the DPS, call Give Me a Break (including web-based communication strategies and web/tablet-based service).<br>• Of particular interest for child-persona construction in this design context are results relating to: (1) pathogenic versus salutogenic perspectives, (2) platforms for Web-based communication, and (3) implications for avatars and conversational user-system interaction.<br>• Multiple sources of data were used for Personas-design (i.e., user group's needs and behaviors were characterized based on cohort data, literature, focus group interviews (with childhood cancer survivors, stakeholder interviews with health care professionals and parents), user interviews, and observations). Data were interpreted and explained together with childhood cancer survivors in 3 explorative design workshops and a validation workshop with children	• The paper present findings and insights on how to co-design child personas in the context of developing digital peer support services with childhood cancer survivors. The work resulted in three primary personas that model the behaviors, attitudes, and goals of three user archetypes tailored for developing health-promoting services in this particular use context.
17.Stasiak et al, 2018 (60)	• **Aim:** Assess feasibility of an online cognitive behavior therapy (CBT) for childhood anxiety<br>• **Methods:** Pragmatic open feasibility pre-post assessment at baseline, 12-week post TX, and 6-month follow-up (n=42)<br>• **Targets:** for aged 7 to 15 children with mild to moderate anxiety after a natural disaster in New Zealand	• BRAVE-ONLINE is an online CBT, adapted from an evidence-based cCBT program (developed by March, et al., 2009; Spence et al., 2008, 2011). It is based on standard CBT anxiety management techniques and come in two versions (for aged 7–12 and for aged 13–18). BRAVE includes 10 sessions (20-45 min) for children; and 5 (in adolescent version) or 6 sessions (in child version) for parents. Parent sessions include parenting strategies (for anxiety management skills and deal with situations when a child gets anxious). If a child or young person misses a session, the system sends out automatic reminders. BRAVE-ONLINE is offered with minimal therapist assistance (estimated on average to be approximately 10 min a week per family). The child completes the program in his/her own time, usually at home, and a therapist reviews the child and parent's input and progress (via an online portal). The therapist uses templates, which they amend accordingly to send personalized feedback to the child and parent separately to support and encourage them to continue. The therapist contacts the family only once by phone in order to coach them in the development of an appropriate individualized exposure hierarchy (called a "BRAVE ladder"). This takes place mid-way through the program and takes approximately 30 min.	• ***Impacts*:** At 6-month post intervention, 55% of the participants assessed, no longer met criteria for their primary anxiety disorder. Participants' anxiety and mood symptoms reduced and health-related quality of life improved significantly by follow-up. Satisfaction ratings were moderate to high (effect on child anxiety & depression was d=.52-.54 at 12-week, and 1.27-1.32 at 6-month follow-up). Those who completed more than four modules had significantly fewer diagnoses at follow-up than those who received a "lower" dose of the program<br>• ***Online session usage***: On average, by 6-month follow-up, children and adolescents had completed 5.9 of 10 sessions (SD=2.9) and parents had completed 4.56/6 (SD=1.7)(child parent program) and 2.95/5 (SD=1.9) sessions (adolescent parent program); 35% and 41% had completed all their sessions at the time of the follow-up.
		**mHealth Literature in School System**	
**18.Pereira, et al., 2015 (61)**	• **Aim:** To develop a web-based teacher education program on childhood mental disorders and evaluate the effectiveness<br>• **Methods:** Cluster RCT with 3 arms: teachers receive web-based interactive education (WBIE, n=52), receiving text and video-based education (TVBE, n=32), and control (n=331)<br>• **Targets:** Primary school teachers in Brazil	• ***WBIE TX:***; a tutorial of the *Web learning management system* (LMS); and *six educational videos* (two address basic and general concepts about common child mental disorder; four provide treatment principles and classroom management guideline based on interview videos with child psychiatrists, teachers, and parents of children with mental disorders); *internet discussion forum*; *Web conference*; and *Written support text*. Participating teachers were also provided with *a booklet* detailing the content presented in the videos.<br>• **TVBE TX (non-web-based)**: the TX is restricted to the tutorial, the videos, and the booklet. The only differences between the WBIE and TVBE programs were the web-based tools used in the former include a discussion forum to interact with consultants and a web conference with a child psychiatrist.<br>• Both TX consisted of 9h of training, delivered in one three-hour session per week for 3 weeks. Teachers can access the program (via online, booklet, and a DVD formats depend on the TX assigned) at anytime	• In terms of gains of **knowledge** about mental disorders, the web-based program TX was superior to the TVBE, and to the control group. The WBIE group was superior to the TVBE group in 4 of the 8 knowledge domains—classroom management; identification of mental health problems; and specific knowledge of depression and conduct disorder. The TVBE group was inferior to the Control group in the specific knowledge of conduct disorder domain<br>• In terms of **beliefs and attitudes** about mental disorders, the WBIE group presented less stigmatized concepts than the TVBE group and more non-stigmatized concepts than the control group. No differences were detected in terms of teachers' attitudes

19.Schuck, et al., 2016 (62)	• **Aim:** Evaluate the feasibility of utilizing iSelfControl, a digital-support TX to improve ADHD children's self-regulation<br>• **Methods:** 13 days of dyadic data from 12 teacher- student pairs over a 6-week period<br>• **Targets:** 9-11 years old with ADHD in the US	• **iSelfControl** is a web-based application designed to support classroom behavior management. iSelfControl prompted students every 30-minute period to self-evaluate focused on compliance, productivity, and positive relationships. Students earned points for demonstrating adaptive behaviors key to school success including: 'Following Directions', 'Following Rules', 'Staying on Task', and 'Getting Along' with others. Demonstrating maladaptive behaviors reduced the number of points a student earned. Simultaneously, the teacher evaluated and recorded behaviors and points earned for each student on a separate iPad.<br>• After logging their entries, students were able to compare their ratings to those made by the teacher, giving them an idea of how consistent their self-evaluation was with that of the teacher. Throughout the day, students can view their progress on charts displayed by the app directly on the iPad	• iSelfControl provides a platform for self and teacher evaluation that is an important adjunct to conventional classroom management strategies.<br>• **Student-teacher consistency**: The results indicate teacher/student discrepancies and significant variations across the day. (e.g., students reported significantly higher mean scores compared to teacher report, but rating for teacher and student rating were relatively stable across days)<br>• **Student variation**: Students varied in self-perception. Some had little insight into the quality of their behavior while other students estimated their performance more harshly than their teacher observed/recorded. This suggests that the application might be more beneficial for some students than for other.
20.Bezem, et al., 2017 (63)	• **Aim:** Study school professionals' perception of the triage approach of School health services (SHS)<br>• **Methods**: a cross-sectional study (comparing the triage (n=444) with the usual approach (n=320)<br>• **Targets:** SHS staff serving 4-8 years old in primary school in Netherlands	• In Netherlands, community-based SHS professionals (physicians, nurse, SHS assistants) usually visit schools a few times a year to carry out routine child health assessments (including using digital tools for behavioral health assessment). The SHS delivers service free of charge for children identified needing additional service<br>• This study examines schools' experience of a routine health assessments based on triage and task-shifting among SHS professionals. In this **two-step triage procedure of preventive care**, pre-assessments (a digital questionnaire) were delegated to SHS assistants who had received specific training (a decision-support function). Only children in need of follow-up were assessed by a SHS physician or nurse, which led to less involvement of physicians and nurses in the routine assessments.	• Respondents from schools using the triage approach had more contacts with SHS and were more satisfied with the appropriateness of support from SHS than respondents in the approach-as-usual group, especially in contribution to the detection of child problems).
**mHealth Literature in Primary-Care System**			
21.Ahlers-Schmidt et al., 2013 (64)	• **Aim:** Asses parents' intention to use the patient portal for their children's health record (EHR) after a facilitated learning session<br>• **Methods:** Cross-sectional (n=171)<br>• **Targets:** Parents of young children from pediatric clinic	• **Patient portal:** is an internet-based self-service model to that allows patients or parents manage health account, information and records (EHR) and be more proactive in their healthcare. A navigator demonstrated the patient portal to parents using on-site kiosks at a pediatric clinic	• Most parents (72%) did not know about the patient portal prior to demonstration; of those who did, only 28% had used<br>• Following demonstration, the majority (92%) thought the patient portal was easy to use. Parents planned to view medical records and laboratory results but disliked having separate accounts for each child and the lack of a ''symptom checker.'' Many (69%) planned for future use.<br>• The majority (97%) found the navigator helpful, and (58%) wanted access to the patient portal via on-site kiosks.
22.Fothergill et al, 2013 (65)	• **Aim:** assess parents and physicians (PCPs) views of a comprehensive, electronic pre-visit screener, and its impact on the visit<br>• **Methods:** a mixed-methods in primary care system (120 parents & 16 PCPs)<br>• **Targets**: Parents and PCPs of 4-10 years old	• The **comprehensive electronic pre-visit screen** includes somatic concerns, health risks, and four mental health tools (SCARED5, PHQ-2, SDQ Impact, and PSC-17). Parents completed the pre-visit screen and an exit survey. PCPs discuss the results with parents during the well-child care visit<br>• A subset of parents was interviewed. All PCPs were interviewed.	• Nearly 90% of parents agreed or strongly agreed that the screener was easy to use and maintained confidentiality. Parents noted that the screener helped with recall, validated concerns, reframed issues they thought might not be appropriate, and raised new questions<br>• PCPs felt that the screener enabled them to normalize sensitive issues, and simultaneously focus and be comprehensive during the visit.<br>• Parents and PCPs agreed that the screener helped guide and promote in-depth discussion, and increased efficiency

23.Glascoe, et al, 2015 (66)	• **Aim:** Provide descriptive patterns about implementation of a web-based pediatric screening PEDStestOnline & clinical recommendations<br>• **Methods:** Data from 79 primary cares (20 states, include 4 settings: general pediatrics/ family medicine; public health departments and community health centers; outpatient teaching-hospital clinics; and others) participated (22 sites participated in in-depth site visit observation/interview<br>• **Targets:** PCPs and parents of 0-8 years-old (20,941 use PEDStestOnline)	• EHRs were in used by > 50% of sites, but EHRs were not integrated with Web-based screening services at any of the sites. Clinicians use online screening and copied/pasted results into EHRs, or, in the case of sites without EHRs, they printed results to include in paper charts.<br>• All participating clinics used **PEDStestOnline** (www.pedstest.com/online), through which clinicians could administer one or more of three evidence-based measures complying with AAP/NAPNAP policies on early detection. PEDStestOnline provides automated scoring, generates referral letters and take-home parent summary reports (in English or Spanish), and identifies appropriate billing and procedure codes. Also available is a parent-portal through which parents can complete measures before the visit but do not see results. Instead, findings are sent to each clinic or provider. Available measures include Parents' Evaluation of Develop-mental Status (PEDS; 10 items), PEDS: Developmental Milestones (PEDS:DM; 6-8 items. available in 25 languages), and Modified Checklist of Autism in Toddlers (M-CHAT; 23 items).	• **Screening tool used**: PEDS was used at 100% of all visits, followed by the PEDS:DM (41%) and the M-CHAT (21%). Use of the M-CHAT spiked around 18months of age and remained high in the months surrounding 24 months of age. The screening use decreased ager 3 years of age.<br>• **Performance**: Prevalence for screening positive (failure rates) was 13% to 16%. Screen at-risk on one or more of the three screens was 22%. Of families completing PEDS, 17% were at high or moderate risk for developmental and/or mental health problems. Of those use PEDS:DM, 17% had two or more unmet milestones, and 8% of those used the M-CHAT received failing scores. Increase in delays as children's age increases (3 years and older were twice more likely to perform poorly on screens than were children 0-2). Also, children who screened outside of the well-child visit schedule (about 34% of children) were 1.5 times higher odds to be at-risk than children screened coinciding with the AAP screening schedule (± 1 month).<br>• **Parent Portal implementation**: In the US, less than 50% clinics implemented web-portal. For the clinics that implemented, % family used portal varied (range of family reached was 35-100%), and only 10% use pre-visit screening. English speaking parents were more likely to use the online portal and screening thank non-English speaking families, but no difference in presence or absence of porta use due to parents' level of education or poverty. *Clinics sign up families for portal in two ways*: i) a waiting room computer kiosk or tablet at check-in (56%); ii) give parents an appointment reminder card, including information on how to log-in to PEDS online & a request to complete screens before the next scheduled visit (44%). *Staffing.* Clinics with computers in waiting rooms often had waiting-room attendants (usually gap year students or retirees paid close to minimum wage) to help parents use computers, probe literacy (by asking if parents preferred assistance), and interview families with limited literacy (e.g., by reading questions aloud). Many of the waiting room attendants were also charged with entertaining children, modeling appropriate adult-child interactions, and in many cases implementing Reach and Read, that is, by reading children's books aloud so that parents could complete screens undisturbed. *Dr. Practice variation/preference*: Some clinicians, once entering the examination room, preferred to begin with PEDS Online screens by interview, as an opening to the encounter. In these cases providers gave live interviews while recording responses onto the PEDS web site. In some clinics, families were scheduled for well-child visit appointments on days when bilingual staff were present.

**24.Farkas, et al, 2015 (30)**	• **Aim:** To identify publicly available education videos on needle pain and anxiety management, and evaluate the content of these videos<br>• **Methods:** A **scoping review** (using YouTube, Google search strategies). The CRAAP Test was used to evaluate the videos quality<br>• **Targets:** Parents & PCPs of infants and toddlers	• 25 relevant educational videos were identified for pain and anxiety generated by needles. The intended audience for most videos was parents (n=16, 64%), followed by clinicians (n=6, 24%) and children (n=3, 12%). Most of the videos were focused on TX for infants and toddlers. Common video examples included immunizations or IV insertion, with interventions appropriate for infants through school-aged children.<br>• The most frequently described techniques in videos were parent-guided distraction (60%), parent behaviors (48%), and child behaviors such as child preparation (32%), breathing (32%), comforting positioning. The length of the videos ranges from 2-24 mins (60% ≤ 6 mins).<br>• Most videos were Current (96%), Relevant (100%), created by a trustworthy source: Authority (76%), and all were Accurate	• No studies have evaluated the effectiveness of these publicly available videos. Strategies for helping older school age children, adolescents, or children with diagnosed needle phobia (15% pediatric population) were not available
25.O'Connor et al., 2016 (67)	• **Aim:** Examine how public health nurses (HVs) engaged and recruited parents in use of eRedBook (EHRs) and associated barriers<br>• **Methods:** Qualitative study(n=33 stakeholders)<br>• **Targets:** HVs serve **p**ediatric population in UK	• **'eRedBook'**, a digitized version of EHRs for parents, was piloted in two National Health Service (NHS) trusts. Public health nurses and Health Visitors (HVs) helped promote the platform to parents, sign them up to use it and document in it as they would the paper-based version.	• Numerous socio-technical barriers affected HVs ability to engage and recruit parents: i) ***technological/EHRs software*** (the complexity of registering and setting up a new profile made it difficult for HVs to enroll parents; parents concern over the security of children's health data; EHRs was not integrated into IT system.); ii) ***Family factors*** (lack of high-speed internet connectivity at home); iii) ***Service Factors***: poor digital literacy skills among HVs. Running electronic & paper form in parallel which doubled HVs workload
26.Singh, et al., 2016 (68)	• **Aim:** Evaluate an EHR-tool for promoting parent ability to identify the care team<br>• **Methods:** pre-post survey (n=61 parents & 63 PCPs)<br>• **Targets:** Parents in the US	• An **EHR-based tool** with names, photos, and definitions of treatment team members (TTMs)	• After use of the improved EHR-TTM system, more parents correctly identified attending physicians by name (71% vs. 28%) and correctly defined terms intern, resident, and attending. Almost all parents (79%) and TTMs (87%) reported that subjects' ability to identify TTMs moderately or strongly impacted satisfaction and trust
27.Cheng et al., 2017 (69)	• **Aim:** Evaluate the impact of an EMR-integrated medical handover tool on staff and pediatric service<br>• **Methods:** Survey with 46 junior medical staff (JMS)<br>• **Targets:** Pediatric care providers in Australia hospitals	• **eHand-over Tool**: An EMR-integrated medical handover tool, to standardize and improve both the standard and efficacy of clinical handover, was developed to improve clinical medical handover between doctors	• ***JMS usage and satisfaction***. A significant uptake of the tool use at 1 and 3-month (87% and 67%) after going-live; and JMS satisfaction with handover process improve (17.4% pre, 80.4% at 1 month, 67.4% at 3 months)<br>• ***Impacts on service system quality & workflow***. The tool significantly reduce paper work time after going-live (6.7% vs. 67.5% users spent less than 15 min updating handover data before and after the implementation of the new tool, 6.7% vs 67.5%); and reduce redundant data (52.2% vs 28.3% with > 25% redundant data before and after eHandover)
28.Destino et al.,, 2017 (70)	• **Aim:** Identify strategies to improve discharge communication in care teams<br>• **Methods:** use Lean A3problem solving methodology with a multidisciplinary team<br>• **Targets:** Pediatric Dr. & PCPs care teams	• **Four strategies** were first identified to improve communication: 1) development of standard process; 2) reduction of overproduction and defects by Making it Easy to Follow the Standard (by providing resident education); 3) eliminating waste in wait and search times (*by improve use of EMR for communication*, e.g., supplying providers with PCP phone number and prefer methods of contact); and 4) aligning the incentive with those performing the work.<br>• Strategies were implemented and then impacts were evaluated (e.g., examining percent PCP call completed or attempted)	• Lean improvement methodology, intensive front-line provider involvement, and process-oriented *electronic health record work flow redesign* led to increased verbal PCP communication at around the time of a patient's discharge.<br>• **Impacts:** E.g., the goal of 80% communication was met and sustained during a 7-month period starting. In the 8-month prior, hospital wide PCP communication prior to discharge averaged 59.1% and improved to 76.7% in the 7-months after

**29.Fiks et al., 2017 (71)**	• **Aim:** Evaluate a distance-learning to improve PCPs use of ADHD rating scales<br>• **Methods:** cluster RCT, with 3 types of learning (19 sites, n=105 PCPs; TX/C=53/52) (assessment at baseline & 8-month follow up)<br>• **Targets:** Pediatric care practices and PCPs	• The **SHARE** intervention was a 3-component distance-learning/quality improvement program***: i) Web-based education*** (three, 15-minute web-based presentations that focused on EBP for managing ADHD in primary care and communication strategies for effectively collaborating with families); ***ii) Collaboratively consultation with ADHD experts*** (via a health system online networking site or private email/telephone conversation); ***iii) Performance feedback reports/calls*** (PCPs received performance feedback reports every 2 months that informed them of their rates of sending and receiving ADHD rating scales from parents and teachers, and allowed them to compare their results to those of the entire group; Feedback reports were discussed during *four, 1-hour conference calls* in which participants reviewed their data and discussed strategies to improve use of EBPs)	• **E-Training**: 79% completed all 3 educational presentations. There were on average two phone, email, or in-person consultations with ADHD experts per month. 30 clinicians participated in at least one performance feedback call (57%). Use of Care Assistant were more frequent in TX (36% use at least 5 times, and 19% use at least 10 times) than in Control (31% use at least 5 time, and 15% use at least 10 times).<br>• **PCPs' Use of ADHD Rating Scale**: Clinicians in both arms showed increase in administering and receiving parent and teacher rating scales. However, there was a moderation TX effect. PCPs in the TX & participated in at least one feedback call or completed all aspects of TX were more likely to give parents rating scales than the TX PCPs who did not participate (14.2% & 18.8% difference)
30.Latif et al., 2017 (72)	• **Aim:** To develop a digital education program for training nurse to improve their knowledge, attitudes and confidence in caring for children with self-harm injuries<br>• **Methods:** using a participatory and co-production approach (n=19)<br>• **Target users:** e-learning target users are nurses	• Nurses, children and young people (CYP) (10-18 year-old) working together to develop the digital education program<br>• **Nurses participated in a priority-setting workshop**. An adapted Delphi technique was used to establish consensus of nurse information needs and educational topic priorities (including exploring nurse experience and challenges; refining initial ideas; and prioritization of topics)<br>• **CYP e-learning development workshop**: CYP (10-18 year-old and had been admitted as an inpatient to acute care services within hospital for the treatment of self-harm injuries within the previous 12 months) were recruited to take part in e-learning development workshop on a weekend. During the workshop, they explore what they thought was important to include in a nurse training package. "Story boards" were used to capture ideas and drawing from the CYP about possible ways to improve the care CYP receive from nurses (e.g., provide personal biographies with life histories to illustrate complex social situations of self-harm and care pathways; culture of openness, respectful collaboration).	• The paper illustrates that involving service users to co-develop educational materials is a feasible and important step in designing educational resources that included a strong patient voice, meet nurse learning needs, and ensures the content is relevant, appropriate and sensitive to both the recipient of care and those responsible for its delivery.

Note. TX=intervention, C=Control. Black bolded references are experimental evaluation studies. Orange bolded references are review articles. PCPs=primary care physicians. References for the 30 reviewed studies (the corresponding reference number 42-72 can be found in the journal article.
